# Progress in Anti-Obesity Drug Development: A Pharmacodynamics Perspective

**DOI:** 10.3390/pharmaceutics18101281

**Published:** 2026-10-09

**Authors:** Ishtiaque Ahmad, Sarfuddin Azmi, Monirah A. Albabtain, Mohammad Mustafa, Faisal Kunnathodi, Riyasdeen Anvarbatcha, Bander R. AlWhaiby, Emtenan M. Alharbi, Amr A. Arafat, Haifa F. Alotaibi

**Affiliations:** 1 Research and Innovation Institute, Ministry of Defense Health Services, Riyadh 12426, Saudi Arabia; iahmad@psmmc.med.sa (I.A.); sazmi@psmmc.med.sa (S.A.); mmustafa@psmmc.med.sa (M.M.); fkunnathodi@psmmc.med.sa (F.K.); ranverbatcha@psmmc.med.sa (R.A.); ba.alwhaiby@modhs.med.sa (B.R.A.); amr.arafat@med.tanta.edu.eg (A.A.A.); 2 Department of Pharmacy, Prince Sultan Cardiac Center, Riyadh 12426, Saudi Arabia; malbabtain@pscc.med.sa; 3 Clinical Pharmacy Department, College of Pharmacy, King Saud University, Riyadh 11451, Saudi Arabia; emtalharbi@ksu.edu.sa; 4 Department of Adult Cardiac Surgery, Prince Sultan Cardiac Center, Riyadh 12426, Saudi Arabia; 5 Department of Family Medicine, Prince Sultan Military Medical City, Riyadh 12426, Saudi Arabia

**Keywords:** anti-obesity medication, obesity pharmacotherapy, pharmacodynamics, GLP-1 agonists, multi-receptor agonists, energy homeostasis, incretin therapy

## Abstract

**Background/Objectives:** Obesity is a chronic, multifactorial condition driven by complex neuroendocrine and metabolic pathways that limit the long-term success of lifestyle interventions. Pharmacotherapy has advanced from early monoaminergic agents to incretin-based and multi-receptor agonists with substantially improved efficacy. This review aims to provide pharmacodynamics-focused synthesis of current and emerging anti-obesity medications, emphasizing mechanistic targets, receptor interactions, and translational implications. **Methods:** We conducted a narrative review integrating Phase I–III clinical trials, mechanistic pharmacology data (receptor affinity, potency, and signaling bias), and regulatory summaries for FDA-approved and selected emerging, investigational, repurposed, or regionally approved anti-obesity agents. Inclusion focused on clinical relevance, pharmacodynamic novelty, and weight-loss efficacy. **Results:** Approved anti-obesity medications act through diverse pathways, including the inhibition of fat absorption (orlistat), the modulation of central appetite circuits (phentermine/topiramate, naltrexone/bupropion), and incretin-based mechanisms (liraglutide, semaglutide, tirzepatide, and the oral GLP-1 receptor agonists semaglutide 25 mg and the nonpeptide orforglipron). GLP-1 receptor agonists achieve approximately 6–15% weight loss, while dual glucose-dependent insulinotropic polypeptide/GLP-1 receptor agonists such as tirzepatide reach 15–20%. Emerging agents—including triple agonists (retatrutide), glucagon-based co-agonists, amylin analogs with Phase III efficacy data, earlier-stage growth differentiation factor 15 (GDF15) pathway agonists, and peripherally restricted cannabinoid-1 receptor inverse agonists—demonstrate enhanced metabolic effects and potential improvements in lean-mass preservation. Obesity-related alterations in pharmacokinetics and pharmacodynamics highlight the need for individualized dosing strategies. **Conclusions:** Clinical efficacy and tolerability cannot be inferred from receptor identity alone. They emerge from relative receptor potency and efficacy, exposure, tissue access, receptor trafficking, and mechanism-linked adverse effects. Dual and triple agonists can broaden efficacy, but receptor balance and standardized comparative pharmacology remain essential for rational development and individualized dosing.

## 1. Introduction

Obesity is a chronic, multifactorial metabolic condition characterized by excessive fat accumulation and a body mass index (BMI) ≥ 30 kg/m^2^. It is a major global health concern, contributing to increased morbidity and mortality through its association with cardiovascular disease, type 2 diabetes mellitus (T2DM), certain cancers, obstructive sleep apnea, osteoarthritis, gallstones, and metabolic syndrome [1,2]. The global prevalence of obesity continues to rise; by 2030, nearly half of adults may be overweight or obese, with obesity specifically (body mass index ≥ 30 kg/m^2^) projected to affect ~22% of women and ~17% of men [3].

Despite the availability of lifestyle-based interventions such as dietary modification, physical activity, and behavioral therapy, long-term weight loss remains elusive for many individuals. This is largely due to the complex neuroendocrine mechanisms that tightly regulate energy homeostasis and defend against negative energy balance [4,5,6,7]. Consequently, pharmacotherapy has emerged as a critical adjunct in obesity management, particularly for individuals with a BMI ≥ 30 kg/m^2^ or ≥27 kg/m^2^ with obesity-related comorbidities [8,9]. The U.S. Food and Drug Administration (FDA) has approved several anti-obesity medications (AOMs), including orlistat, phentermine-topiramate, naltrexone-bupropion, liraglutide, semaglutide, tirzepatide, and oral semaglutide 25 mg—approved in December 2025 as the first oral GLP-1 receptor agonist for chronic weight management—and orforglipron (Foundayo), which received FDA approval on 1 April 2026 [10,11]. These agents act via central and/or peripheral mechanisms to reduce appetite, enhance satiety, or inhibit nutrient absorption, resulting in clinically meaningful weight loss of 5–15% when combined with lifestyle interventions [12,13,14]. Key pharmacodynamic parameters, including receptor affinity (KD/Ki), potency (EC_50_), pathway-specific signaling, and tissue-specific engagement for major drug classes, are summarized in Section 5.

Pharmacological strategies have evolved from early monoaminergic agents with limited efficacy and safety to newer therapies that target specific neurohormonal pathways. These include inhibition of pancreatic lipases (e.g., orlistat) to reduce fat absorption [15], the activation of glucagon-like peptide-1 (GLP-1) receptors (e.g., liraglutide, semaglutide) to enhance satiety [16], the modulation of leptin signaling [17], and the stimulation of melanocortin-4 receptors (MC4R) for central energy regulation [7,18]. However, despite the availability of these agents, barriers such as cost, limited insurance coverage, tolerability issues, and the perception of obesity as a behavioral rather than medical condition continue to hinder their widespread use.

Obesity can also alter gut permeability, gastric emptying, cardiovascular function, and hepatic and renal physiology, further complicating PK/PD responses. From a PD standpoint, obesity may modify target-receptor density and coupling efficiency (e.g., central satiety circuits, GLP-1R/GCGR/GIPR signaling), altering exposure–response curves even when systemic exposure is unchanged, underscoring the need to evaluate both PK and PD when dosing in severe obesity [19]. These alterations can affect drug absorption, distribution, metabolism, and excretion, necessitating individualized dosing strategies, especially in patients with severe obesity (BMI > 35–40 kg/m^2^) [20,21]. Obesity-induced chronic inflammation and adipokine dysregulation (e.g., leptin resistance) further complicate drug response and safety profiles [20,22].

Despite these complexities, the underrepresentation of obese patients in clinical trials has led to a scarcity of evidence-based dosing recommendations [23], leaving most Summaries of Product Characteristics without specific guidance. Moreover, a primary focus on pharmacokinetics alone is insufficient for developing optimized dosing regimens, as it fails to account for the complex pharmacodynamic changes associated with obesity. This gap underscores the need for more inclusive research and the development of physiologically based PK/PD models to guide personalized dosing [24]. A deeper understanding of pharmacodynamics is essential not only for optimizing drug efficacy and safety but also for addressing the long-standing challenges in obesity treatment, including poor adherence, weight regain after discontinuation, and limited long-term success with pharmacotherapy.

**Figure 1:**
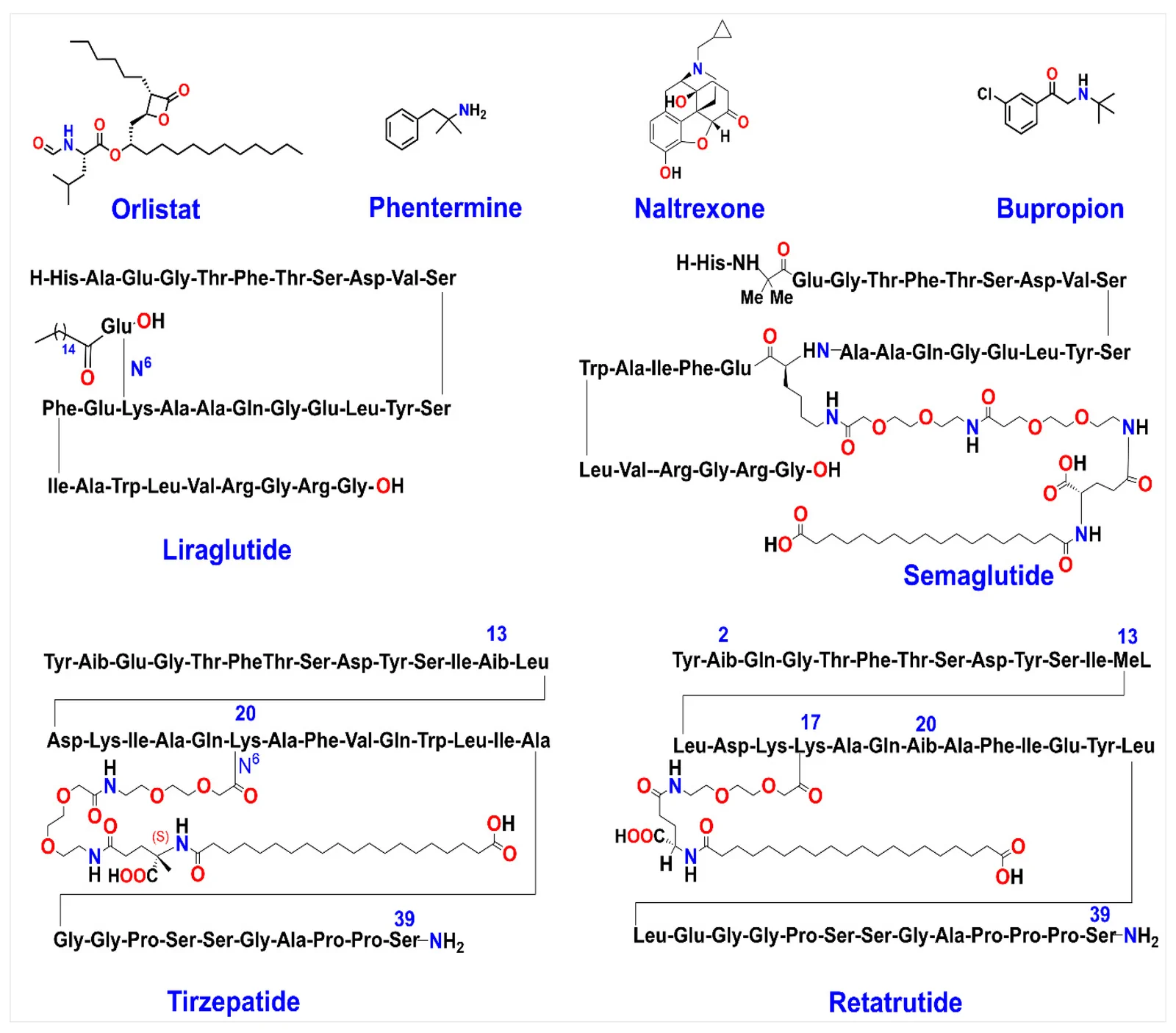
Representative structural frameworks of approved and investigational anti-obesity agents. Small-molecule agents are shown together with peptide-based incretin and multi-receptor agonists. The peptide structures illustrate amino-acid substitution and lipidation strategies used to increase enzymatic stability, albumin binding, duration of receptor exposure, and receptor selectivity. Aib denotes alpha-aminoisobutyric acid; H-His denotes histidine with a free N-terminal amino group; and MeL denotes alpha-methyl-L-leucine (alpha-Me-Leu), the noncanonical residue at position 13 of retatrutide [26]. The lipid side chains shown for liraglutide, semaglutide, tirzepatide, and retatrutide prolong systemic exposure. Retatrutide is investigational; all other agents shown are FDA-approved or components of FDA-approved combinations.

This review provides a pharmacodynamics-focused synthesis of anti-obesity therapies, from single-target agents to multi-receptor agonists. Unlike previous reviews organized mainly by clinical efficacy and safety [1,13,15,25], it uses receptor pharmacology as its framework, separating affinity, potency, and clinical exposure; distinguishing quantified signaling bias from pathway preference; and linking exposure to efficacy, gastrointestinal tolerability, and dose titration. Figure 1 illustrates representative structures and peptide-modification strategies of incretin-based therapies.

## 2. Materials and Methods

This narrative review synthesizes evidence from peer-reviewed clinical trials, mechanistic studies, and regulatory documents related to FDA-approved and selected emerging, investigational, repurposed, or regionally approved anti-obesity agents. PubMed, Scopus, and Google Scholar were searched from inception to July 2026, supplemented by hand-searched reference lists, ClinicalTrials.gov, and regulatory resources; search terms and extraction fields are detailed in Supplementary Material S1. Sources were selected purposively; consistent with the narrative design, no PRISMA flow diagram was produced, and selection may be incomplete. FDA-approved agents were included irrespective of the following criteria. other agents were included if they met at least one of the following a priori criteria: (i) at least approximately 5% weight loss in controlled human studies, the threshold generally regarded as clinically meaningful [8,9]; (ii) mechanistic novelty (e.g., multi-agonism or amylin, ActRII, or GDF15 pathways); or (iii) translational pharmacology data linking receptor engagement to clinical outcomes. Criteria were applied disjunctively; agents meeting only criterion (ii) or (iii) were retained even where reported weight loss was below 5% (for example, LY3463251 and INV-202) because their value here is mechanistic rather than efficacy-based. Regulatory status as of July 2026 was classified as FDA-approved for obesity (A), regionally approved for obesity (R), repurposed from another approved indication (Rp), or investigational (I, with highest phase); this classification is applied in Table 1, Table 2 and Table 3 and Figure 1.

### Targeted Pharmacodynamic Search and Data Extraction

To address receptor-level pharmacology specifically, a focused search was updated through July 2026 using each included drug or target name combined with KD, Ki, EC_50_, IC_50_, Emax, receptor occupancy, cAMP, beta-arrestin, internalization, signaling bias, off-target, tissue distribution, or exposure–response. Primary receptor-pharmacology and human PK/PD studies were prioritized over narrative summaries. For each source, we extracted receptor species or subtype, expression system or tissue, assay and readout, comparator ligand, quantitative affinity or potency, maximal response, trafficking or pathway data, tissue distribution, and documented secondary targets. Values were not pooled across heterogeneous assays; ranges were used only for measurements sharing receptor species, expression system, and assay readout. Because this is a narrative review, the targeted search is presented as a transparent source-specific synthesis rather than a systematic review or meta-analysis.

## 3. Neuroendocrine Basis of Obesity and Rationale for Pharmacological Targeting

Because pharmacodynamic effects of anti-obesity drugs are rooted in the biology of energy homeostasis, we first outline the neuroendocrine circuits relevant to receptor–drug interactions (GLP-1R, GIPR, GCGR, MC4R, CB1R, amylin). This mechanistic primer also provides the context for interpreting the PD properties (affinity/potency, signaling bias) discussed in the therapeutic sections. Obesity is increasingly recognized as a neuroendocrine disorder resulting from dysregulation of central appetite control and peripheral metabolic signaling rather than solely an imbalance between caloric intake and expenditure. Understanding the neuroendocrine architecture governing energy homeostasis is therefore essential for interpreting the pharmacodynamic rationale of contemporary anti-obesity therapies [4].

## 4. Pharmacologically Relevant Neural Regulation of Energy Homeostasis

Neural regulation is included here only where it explains drug–target pharmacodynamics. The arcuate nucleus integrates peripheral nutrient and hormonal signals through orexigenic AgRP/NPY neurons and anorexigenic POMC/CART neurons. POMC-derived alpha-melanocyte-stimulating hormone activates MC4R to reduce food intake and increase autonomic energy expenditure; this downstream position explains why the MC4R agonist setmelanotide can restore signaling in selected upstream melanocortin-pathway defects [25,27,28,29,30].

Gut–brain pharmacology is mediated through both circulating ligands and vagal or brainstem pathways. GLP-1R and amylin-receptor activation in the area postrema, nucleus tractus solitarius, and hypothalamic circuits reduce meal size and promote satiation, while delayed gastric emptying contributes to early satiety and dose-limiting gastrointestinal effects. GIPR engagement adds insulinotropic and adipose-tissue actions, whereas GCGR engagement acts predominantly in liver to increase substrate mobilization and energy expenditure [31,32,33,34,35].

The distinction between central and peripheral target engagement is clinically important. Central CB1R blockade can reduce appetite but caused unacceptable psychiatric adverse effects with earlier agents, whereas peripherally restricted CB1R inverse agonists are designed to retain hepatic and adipose metabolic effects while minimizing brain exposure. This tissue-selective strategy is a pharmacodynamic design principle rather than a general review of reward neurobiology [36,37,38].

Astrocytic and microglial modulation of hypothalamic signaling and mesolimbic reward circuitry provide additional biological context for appetite control; however, these pathways are not reviewed further here because direct receptor-level evidence linking them to current anti-obesity drug dosing remains limited [39,40].

Modern anti-obesity therapies therefore act on defined receptor systems rather than on neural circuits in the abstract. GLP-1R agonists reduce hunger through gut–brain and hypothalamic pathways; tirzepatide integrates GIPR and GLP-1R signaling; retatrutide adds GCGR-mediated metabolic activity; naltrexone/bupropion sustains POMC signaling and modifies reward-related eating; and setmelanotide directly activates MC4R. Figure 1 summarizes the chemical scaffolds and peptide modifications that support these receptor-level effects [30,41,42,43,44].

Accordingly, the therapeutic sections below focus on the sequence from receptor engagement to intracellular signaling, tissue response, clinical dose–response, efficacy, and adverse effects. GLP-1R, GIPR, MC4R, and amylin-receptor pathways primarily influence satiation and feeding behavior, whereas GCGR and peripheral CB1R pathways contribute more directly to hepatic metabolism, adipose-tissue lipid turnover, and energy expenditure [30,45,46].

### Cellular Pathways (Intracellular Signaling)

For the principal peptide targets, extracellular ligand binding is translated into intracellular responses through receptor-specific coupling. GLP-1R, GIPR, and GCGR are class B G-protein-coupled receptors that predominantly couple to stimulatory G proteins (Gs), activating adenylyl cyclase, cyclic adenosine monophosphate (cAMP), protein kinase A, and exchange proteins directly activated by cAMP. In pancreatic beta cells, GLP-1R and GIPR signaling amplifies glucose-dependent insulin secretion; in neuronal circuits it supports satiation, while hepatic GCGR signaling promotes glycogenolysis, lipid oxidation, and energy expenditure. MC4R also signals mainly through Gs-cAMP, although cell context can permit additional Gq/phospholipase-C signaling. Amylin receptors are calcitonin-receptor/receptor-activity-modifying protein complexes whose cAMP potency and tissue response depend on receptor subtype and cellular background [11,18,34,35,47].

Not all anti-obesity targets use the same intracellular architecture. CB1R is predominantly Gi/o-coupled; constitutive or ligand-induced CB1R activity reduces adenylyl-cyclase activity and modulates extracellular signal-regulated kinase, whereas inverse agonism suppresses this signaling. GDF15 analogs such as LY3463251 bind GFRAL and recruit the RET receptor tyrosine kinase, producing downstream ERK/MAPK and AKT signaling in brainstem neurons. ActRII ligands signal through SMAD2/3, so receptor blockade or ligand trapping shifts the balance away from activin/myostatin-mediated catabolic signaling. By contrast, orlistat and EMP-16 act through digestive-enzyme inhibition in the gastrointestinal lumen; GPCR signaling and signaling-bias terminology do not apply to these agents [37,38,48,49,50,51].

Pathway preference must be separated from a formal quantitative bias factor. Formal bias quantification requires full concentration–response curves for at least two pathways, a common reference agonist, and matched receptor density, cellular background, assay timing, and analysis, typically using an operational-model ΔΔlog(τ/KA) estimate [52]. No directly comparable numerical bias factor generated with matched pathways, a common reference ligand, and the same receptor and cell system was available across the major clinical candidates. Quantitative bias estimates have been reported for some individual ligands, including GLP-1R/GCGR dual agonists, but not on a basis that permits cross-candidate comparison [19,52]. We therefore report source-derived pathway metrics without calculating or reporting an unsupported bias value. Ecnoglutide showed a cAMP EC50 of 0.018 nM and an internalization EC50 above 10,000 nM, representing a greater than 5.5 × 10^5^-fold within-study potency separation. At GLP-1R, tirzepatide showed a Gs EC_50_ of 0.617 nM and an internalization EC_50_ of 101.9 nM (approximately 165-fold separation), with internalization Emax of 43.6% of GLP-1 and beta-arrestin efficacy below 10% [53,54]. These comparisons demonstrate pathway preference but are not substitutes for a formal operational-model bias factor; the distinction prevents receptor reserve or assay amplification from being misinterpreted as ligand bias. Whether such pathway preference yields clinically meaningful differences in weight loss or gastrointestinal tolerability in humans remains unestablished because comparisons at matched exposure are lacking.

## 5. Current Anti-Obesity Medications: Targeting Peripheral and Central Nervous Systems

Food and Drug Administration-approved anti-obesity medications primarily reduce energy intake by modulating appetite, satiation, or nutrient absorption. These agents act through central or peripheral mechanisms and are typically prescribed alongside lifestyle interventions [8,12,13]. Table 1 summarizes clinical mechanisms and therapeutic implications, whereas Table 2 provides receptor-level affinity, potency, assay context, intracellular signaling, tissue distribution, and available quantitative pathway-bias information.

**Table 1:** FDA-approved anti-obesity medications: mechanisms of action, efficacy, and safety considerations.

Medication/FDA Approval	Mechanism of Action and Pharmacodynamic Effects	Clinical Implication and Efficacy	Safety Considerations, Contraindications, and Important Precautions
Phentermine (Adipex-P, Lomaira) [8,55,56,57,58] FDA approval: 1959	Sympathomimetic amine Increases hypothalamic norepinephrine release Reduces appetite and food intake	Clinical implication: Approved for short-term use only (≤12 weeks); Schedule IV; tachyphylaxis limits chronic use. Average weight loss: ~6.1% at 28 weeks with phentermine 15 mg vs. 1.7% with placebo (46.2% vs. 15.5% achieved ≥ 5%)	Common adverse effects: Dry mouth, insomnia, constipation, and increased heart rate. Contraindications: Cardiovascular disease, uncontrolled hypertension, hyperthyroidism, glaucoma, agitated states, drug-abuse history, monoamine oxidase inhibitor use, and pregnancy. Important precaution: Monitor heart rate and blood pressure.
Orlistat (Xenical) [59,60] FDA approval: 1999	Gastric and pancreatic lipase inhibitor • Blocks triglyceride breakdown • Reduces dietary fat absorption in the intestines	Clinical implication: Non-systemic lipase inhibitor; reduces dietary fat absorption by approximately 30%; counsel patients on fat-soluble vitamin supplementation. Average weight loss: −5.8 vs. −3.0 kg PBO, 4 y (XENDOS; ITT)	Common adverse effects: Gastrointestinal disturbance, diarrhea, abdominal bloating, and deficiency of fat-soluble vitamins. Contraindications/important precautions: Chronic malabsorption syndrome, cholestasis, and pregnancy.
Phentermine/Topiramate (Qsymia) [55,56,61,62,63,64] FDA approval: 2012	Phentermine is a sympathomimetic amine that enhances hypothalamic catecholamine signaling, reducing appetite and food intake. Topiramate augments GABAergic activity, modulates voltage-gated ion channels, antagonizes AMPA/kainate glutamate receptors, and inhibits carbonic anhydrase, contributing to appetite suppression and enhanced satiety.	Clinical implication: Highly effective oral combination; teratogenic risk requires REMS. Average weight loss: −7.8% (7.5/46 mg), −9.8% (15/92 mg) vs. −1.2% PBO, 56 wk (CONQUER; ITT; included T2D)	Common adverse effects: Paresthesia, dry mouth, gastrointestinal disturbance, depression, and insomnia. Contraindications/important precautions: Pregnancy, glaucoma, hyperthyroidism, monoamine oxidase inhibitor use, and hypersensitivity to sympathomimetic amines.
Naltrexone/bupropion (Contrave, Mysimba) [65,66,67] FDA approval: 2014	Opioid receptor antagonist plus dopamine and norepinephrine reuptake inhibitor • Appetite suppression in the hypothalamus • Modulation of the mesolimbic reward system to reduce food cravings	Clinical implication: Targets reward-driven eating; avoid uncontrolled HTN/seizure risk Average weight loss: −6.1% (32/360 mg) vs. −1.3% PBO, 56 wk (COR-I; mITT, LOCF)	Common adverse effects: Nausea, headache, constipation, and vomiting. Contraindications/important precautions: Seizure disorders, uncontrolled hypertension, eating disorders, chronic opioid use or dependence, monoamine oxidase inhibitor use, and pregnancy.
Liraglutide (Saxenda) [68,69] FDA approval: 2014	GLP-1 receptor agonist • Appetite suppression • Increased satiety • Slowed gastric emptying	Clinical implication: Incremental efficacy; GI AEs; dose titration improves tolerability Average weight loss: −8.4 vs. −2.8 kg PBO, 56 wk (SCALE; LOCF)	Common adverse effects: Nausea, diarrhea, constipation, and dyspepsia. Contraindications: Personal or family history of medullary thyroid carcinoma (MTC), Multiple Endocrine Neoplasia syndrome type 2 (MEN 2), or serious hypersensitivity. Important precaution: Discontinue when pregnancy is recognized.
Setmelanotide (Imcivree) * [30,70,71,72] FDA approval: 2020	Melanocortin-4 receptor (MC4R) agonist • Activates the MC4 pathway to restore impaired melanocortin signaling • Increases energy expenditure • Reduces hunger and increases satiety	Clinical implication: Indicated for acquired hypothalamic obesity in patients aged ≥ 4 years and for Bardet–Biedl syndrome or POMC/PCSK1/LEPR deficiency in patients aged ≥ 2 years; not indicated for general (polygenic) obesity. Efficacy: Indication-specific; in the pivotal acquired hypothalamic obesity trial, in the pivotal acquired hypothalamic obesity trial (TRANSCEND), mean BMI change at 52 weeks was −16.5% with setmelanotide versus + 3.3% with placebo, a placebo-adjusted difference of −19.8 percentage points (*p* < 0.001).	Common adverse effects: Skin hyperpigmentation, injection-site reactions, nausea, headache, diarrhea, abdominal pain, vomiting, depression, and spontaneous penile erection. Contraindication: Prior serious hypersensitivity to setmelanotide or its excipients. Important precautions: Sexual adverse reactions, depression/suicidality, and skin-pigmentation changes; in acquired hypothalamic obesity, monitor adrenal function and sodium balance when clinically relevant. Discontinue when pregnancy is recognized.
Semaglutide, subcutaneous (Wegovy) [12,45] FDA approval: 2021	GLP-1 receptor agonist • Significant appetite suppression • Increased satiety • Delayed gastric emptying	Clinical implication: High-efficacy GLP-1RA; requires dose titration for GI tolerability; sustained weight loss with continued therapy. Average weight loss: −14.9% vs. −2.4% PBO, 68 wk (STEP 1; TP)	Common adverse effects: Nausea, vomiting, and diarrhea. Contraindications: Personal or family history of MTC, MEN 2, or serious hypersensitivity. Important precaution: For weight reduction, discontinue when pregnancy is recognized.
Oral semaglutide (Wegovy tablets) [73,74] FDA approval: December 2025	GLP-1 receptor agonist (oral peptide) • Appetite suppression • Increased satiety • Delayed gastric emptying • SNAC absorption enhancer enables oral bioavailability	Clinical implication: First oral GLP-1RA for weight management; requires fasted dosing with ≤120 mL water and a 30 min wait. Average weight loss: −13.6%, 64 wk (TP; −16.6% EF)	Boxed warning: Thyroid C-cell tumors. Common adverse effects: Nausea, vomiting, diarrhea, and constipation. Contraindications: Personal or family history of MTC, MEN 2, or serious hypersensitivity. Important precautions: Delayed gastric emptying may alter absorption of concomitant oral drugs. Discontinue when pregnancy is recognized.
Tirzepatide (Zepbound) [75,76] FDA approval: 2023	GIP/GLP-1 dual agonist • Enhanced appetite suppression • Increased satiety • Delayed gastric emptying	Clinical implication: Greatest mean weight loss among approved agents; continued therapy needed to sustain effect Average weight loss: −15.0% to −20.9% (5–15 mg) vs. −3.1% PBO, 72 wk (SURMOUNT-1; TP)	Common adverse effects: Nausea, diarrhea, dyspepsia, abdominal pain, and reduced appetite. Contraindications: Personal or family history of MTC, MEN 2, or serious hypersensitivity. Important precaution: Discontinue when pregnancy is recognized.
Orforglipron (Foundayo) [10,77,78,79] FDA approval: 2026	Oral, nonpeptide GLP-1 receptor partial agonist • Activates GLP-1R/cAMP signaling • cAMP-preferring; minimal beta-arrestin recruitment and limited internalization; human GLP-1R Ki ≈ 1 nM • Suppresses appetite and increases satiety • Delays gastric emptying	Clinical implication: Once-daily oral small-molecule GLP-1RA; can be administered without an empty-stomach requirement; gradual dose escalation manages on-target gastrointestinal tolerability. Average weight loss: −7.8% to −12.4% vs. −0.9% PBO, 72 wk (EF; −7.5% to −11.2% TP)	Common adverse effects: Nausea, diarrhea, constipation, vomiting, abdominal pain, and dyspepsia. Contraindications: Personal or family history of MTC, MEN 2, or serious hypersensitivity. Important precautions: Avoid coadministration with another GLP-1 receptor agonist; monitor for pancreatitis, severe gastrointestinal reactions, volume depletion, gallbladder disease, and hypoglycemia when used with insulin or an insulin secretagogue. Discontinue when pregnancy is recognized.

Footnote: Weight-loss values come from individual trials differing in population, duration, background intervention, and estimand; they are mean changes from baseline unless stated as placebo-adjusted and should not be compared across rows. Weight loss values are reported as approximate mean percentage change from baseline body weight at one year unless otherwise specified. Trade names are presented according to FDA- and EMA-approved nomenclature; regional brand name differences are indicated where applicable. Abbreviations used in Table 1: adverse events (AEs); efficacy/trial-product estimand (EF); gastrointestinal (GI); glucagon-like peptide-1 receptor agonist (GLP-1RA); hypertension (HTN); intention to treat (ITT); last observation carried forward (LOCF); modified intention to treat (mITT); monoamine oxidase inhibitor (MAOI); placebo (PBO); Risk Evaluation and Mitigation Strategy (REMS); treatment-policy/treatment-regimen estimand (TP); type 2 diabetes (T2D). Detailed receptor pharmacology is summarized in Table 2. Regulatory scope of Table 1: Retatrutide, which remains investigational, is discussed in Section 9.3 and Table 3 [80,81]. Metreleptin is discussed in Section 13 because its approved indication is replacement therapy for complications of leptin deficiency in generalized lipodystrophy rather than common obesity. Gelesis100 (Plenity) is discussed in Section 5.2 as an FDA-authorized oral hydrogel medical device rather than as an anti-obesity medication [82,83]. * Approved only for obesity due to specific genetic MC4R-pathway disorders and acquired hypothalamic obesity.

### 5.1. Interpretation of Pharmacodynamic Parameters and Assay Context

Binding affinity and functional potency describe different properties. KD and Ki quantify ligand-target binding under defined experimental conditions, whereas EC_50_ and IC_50_ describe the concentration producing half-maximal activation or inhibition in a particular functional assay. A lower numerical value generally indicates greater affinity or potency within the same assay, but values cannot be ranked across studies without considering receptor species and subtype, expression density, cell background, pathway readout, incubation time, albumin content, and receptor reserve. The same ligand may therefore produce different EC_50_ values in cAMP, beta-arrestin, internalization, calcium, ERK, or transcriptional assays [19,52]. Efficacy (Emax), the maximal achievable response, differs from potency; tirzepatide, for example, is potent but partial at GLP-1R (51% Emax) [54]. Receptor occupancy depends on affinity and free-drug exposure, and receptor reserve allows maximal response at submaximal occupancy [52].

Clinical dose is not determined by in vitro potency alone. Protein binding, absorption, clearance, free-drug concentration, tissue access, target density, and the duration of receptor occupancy determine whether an in vitro potency difference translates into greater weight loss. For lipidated peptides, albumin binding prolongs exposure but also changes the free concentration available to engage receptors. Dose escalation of GLP-1R-containing regimens consequently reflects both the exposure needed for sustained central and peripheral target engagement and the need to accommodate on-target gastrointestinal pharmacology [54,84,85,86,87].

Table 2 reports values as source-specific ranges or representative values rather than as a single cross-study potency ranking. It also distinguishes formal bias factors from descriptive pathway preference. Where peer-reviewed receptor-level values were not reported for a clinical candidate, the table states this explicitly rather than imputing a value from a different ligand, assay, or receptor system.

Across FDA-approved anti-obesity medications, several consistent physiological outcomes complement their primary pharmacodynamic mechanisms. Incretin-based agents such as liraglutide, semaglutide, tirzepatide, and orforglipron reduce hunger and overall energy intake through central and peripheral GLP-1R-containing pathways [10,68,75,77]. Centrally acting combinations such as phentermine/topiramate and naltrexone/bupropion predominantly reduce appetite or reward-driven eating, whereas orlistat reduces fat absorption. Gelesis100 is considered separately because it is a non-systemic oral hydrogel medical device that promotes fullness through gastric expansion rather than receptor-mediated pharmacology [59,60,83]. Across pharmacologic classes, reductions in fat mass are accompanied by improvements in insulin sensitivity, lipid parameters, and systemic inflammation, with GLP-1-based therapies generally producing the most favorable fat-to-lean-mass profile [12,45,75].

**Table 2:** Receptor-level pharmacodynamic parameters, cellular signaling, and assay context for major anti-obesity drug classes.

Drug/Class	Target and Testing Environment	Quantitative PD Values	Cellular Pathway/Bias Interpretation	Tissue and Clinical Interpretation	Refs.
GLP-1R agonists: liraglutide (A), semaglutide (A),	Human GLP-1R in BHK/HEK293 expression systems; radioligand binding, cAMP assays.	Liraglutide and semaglutide show human GLP-1R cAMP EC50 values in the low-picomolar range (approximately 0.01–0.03 nM); semaglutide binding IC50 approximately 0.38 nM. Liraglutide binding 0.11 nM.	Predominantly Gs-cAMP signaling.	Pancreas, gut, vagal/brainstem and hypothalamic circuits. Lipidation prolongs exposure; dose escalation balances sustained receptor engagement against on-target nausea, vomiting, and delayed gastric emptying.	[84,86,87]
Ecnoglutide (R)	Human GLP-1R in [BHK/HEK293] cells; cAMP and internalization assays	cAMP EC50 0.018 nM; internalization EC50 > 10,000 nM.	Very large separation between cAMP potency and internalization; this supports cAMP pathway preference, but a formal ΔΔlog(τ/K_A)value requires matched reference-ligand pathway analysis.	As for liraglutide and semaglutide.	[53]
Tirzepatide (A)	Low-density human GIPR- or GLP-1R-expressing HEK293 cells for cAMP/Gs assays; CHO-K1 cells for beta-arrestin recruitment and receptor internalization.	GIPR Gs EC50 0.379 nM with full efficacy; GLP-1R Gs EC_50_ 0.617 nM with 51% Emax. GIPR beta-arrestin-2 EC50 2.34 nM; GLP-1R beta-arrestin efficacy < 10%. GLP-1R internalization EC_50_ 101.9 nM and Emax approximately 43.6% of GLP-1.	Full agonism resembling native GIP at GIPR, with greater overall GIPR engagement. At GLP-1R, tirzepatide is a partial agonist with cAMP preference over beta-arrestin recruitment and approximately 165-fold separation between Gs and internalization potency. This is quantitative pathway preference at GLP-1R; a formal operational-model bias factor was not reported.	Integrated pancreatic, adipose, and central effects. Greater GIPR engagement permits clinically meaningful dual-receptor activity while GLP-1R-mediated gastrointestinal effects remain a constraint on dose escalation.	[54]
Retatrutide (I)	Human GIPR, GLP-1R, and GCGR expression systems; binding and cAMP concentration–response assays performed separately for each receptor.	Human cAMP EC_50_: GIPR 0.0643 nM, GLP-1R 0.775 nM, and GCGR 5.79 nM. Reported human binding Ki: GIPR 0.057 nM, GLP-1R 7.2 nM, and GCGR 5.6 nM.	Greatest potency at GIPR; GLP-1R versus GCGR ordering differs between cAMP and binding data, so ranking is approximate. GIPR/GLP-1R activity combine insulinotropic/satiety effects with a glucagon component that can increase energy expenditure.	Pancreas and central feeding pathways through GIPR/GLP-1R, with hepatic GCGR engagement. Increasing GCGR contribution may improve fat oxidation but can contribute to heart-rate and tolerability signals.	[26]
Survodutide (BI 456906) (I)	CHO-K1 cells expressing human GLP-1R or human GCGR; functional cAMP-response assays. Mouse endogenous GLP-1R also assessed in MIN6 cells.	Human GLP-1R EC_50_ 0.33 nM and human GCGR EC50 0.52 nM; native GLP-1 and glucagon were more potent in the same system (approximately 0.06 and 0.02 nM).	Dual Gs-cAMP agonism with similar subnanomolar potency at the two human receptors in the reported assay. No formal cross-pathway bias factor was reported.	GLP-1R contributes appetite suppression and glucose control; hepatic GCGR contributes substrate mobilization and energy expenditure. Therapeutic activity depends on receptor balance rather than maximal activation of either receptor alone.	[88]
Cagrilintide (I)	Human AMY3R and calcitonin receptor luciferase assays; rat AMY3R and calcitonin-receptor cAMP assays; receptor composition includes calcitonin receptor plus RAMP3.	Human functional EC_50_: AMY3R 49 pM and calcitonin receptor 62 pM. Human binding IC_50_: AMY3R 170 pM and calcitonin receptor 233 pM. Rat cAMP EC_50_: AMY3R 348 pM and calcitonin receptor 287 pM.	Long-acting amylin-receptor agonism. Similar potency at AMY3R and calcitonin receptor means that receptor-subtype composition and tissue context must be reported; a generalized Gs-bias claim is not supported by these data alone.	Area postrema and other brainstem/hypothalamic satiation circuits, with gastrointestinal effects. Lipidation prolongs exposure and reduces meal size, but nausea remains an on-target limitation.	[11]
Setmelanotide (A) *	Human MC4R binding and cAMP assays in transfected cell systems; comparator studies also assessed MC1R, MC3R, MC5R, and alternative NFAT/PLC readouts.	Reported human MC4R Ki approximately 0.71–2.1 nM and cAMP EC_50_ approximately 0.27–1.5 nM. Off-target activation is weaker: MC1R EC_50_ approximately 5.8 nM and MC3R approximately 5.3 nM; NFAT/PLC EC_50_ approximately 5.9 nM in a separate system.	Potent MC4R Gs-cAMP activation with evidence of additional Gq/PLC signaling. Formal bias depends on matched assays. The receptor-selectivity window is clinically relevant because MC1R cross-activation explains hyperpigmentation.	Hypothalamic melanocortin circuits; approved for acquired hypothalamic obesity and selected Bardet–Biedl/POMC/PCSK1/LEPR deficiency, not general obesity. Off-target MC1R activity occurs in skin.	[18,47,89]
Orlistat (A) and EMP-16 (I)	Gastric/pancreatic lipase assays and intestinal alpha-glucosidase assays; enzyme source, substrate, formulation, and exposure time materially affect IC_50_.	Orlistat IC_50_ values vary across lipase preparations and assay substrates and should not be pooled as a receptor potency. The clinical EMP-16 report did not provide peer-reviewed component-specific alpha-glucosidase and lipase IC_50_ values.	Covalent lipase inhibition by orlistat; EMP-16 combines local lipase inhibition with acarbose-mediated alpha-glucosidase inhibition. GPCR signaling, beta-arrestin recruitment, and signaling bias are not applicable.	Predominantly gastrointestinal-luminal action with minimal systemic exposure. Clinical effects and adverse events reflect reduced fat/carbohydrate digestion, including steatorrhea, diarrhea, and bloating.	[15,48,90,91]
LY3463251 (I)	Surface-plasmon-resonance binding and ERK-phosphorylation assays in cells overexpressing human or animal GFRAL together with RET.	Human GFRAL binding Kd 3.1 nM; human GFRAL/RET ERK-phosphorylation EC_50_ 54.7 nM. Mouse functional EC_50_ 136.2 nM was reported separately and was not pooled with the human value.	GDF15 analog fused to monomeric IgG4-Fc. GFRAL recruits RET receptor tyrosine kinase and activates ERK/MAPK and AKT signaling. This is not GLP-1R signaling and is not a GPCR-bias mechanism.	GFRAL expression is concentrated in the area postrema and nucleus tractus solitarius. Reduced food intake and appetite are accompanied by dose-related nausea/emesis in some participants.	[50]
Maridebart cafraglutide (MariTide, AMG 133) (I)	Human GLP-1R agonist cAMP assays and human GIPR antagonist assays; long-acting peptide–antibody conjugate.	Reported GLP-1R agonist EC_50_ 24.4 pM and GIPR antagonist IC_50_ 42.4 nM in the development program.	Combines GLP-1R Gs-cAMP agonism with functional GIPR blockade. This mechanism is pharmacologically distinct from tirzepatide, which agonizes both GIPR and GLP-1R.	Systemic, prolonged exposure supports intermittent dosing. Weight-loss effects reflect GLP-1R-mediated appetite reduction plus GIPR antagonism; gastrointestinal tolerability remains linked to GLP-1R activity.	[92]
INV-202 (I)	Peripherally restricted CB1R inverse agonist; clinical paper reports metabolic outcomes but no complete receptor-level Ki/EC_50_ panel.	No comparable receptor-level potency range was available in the cited clinical reports; values from unrelated CB1 ligands or different ActRII biologics were not substituted.	Suppresses peripheral Gi/o-CB1R signaling.	Peripheral CB1R restriction is intended to avoid psychiatric effects of central CB1 blockade.	[37,38]
bimagrumab (I)	ActRII-targeting monoclonal antibody.	Not reported; values from different ActRII biologics were not substituted	Blocks ActRII signaling and reduces SMAD2/3 activation downstream of activin/myostatin.	ActRII blockade acts mainly in skeletal muscle and adipose tissue, shifting body composition toward greater lean mass.	[49]

Note: Values are assay-specific and should not be interpreted as direct head-to-head rankings. Formal pathway bias requires matched pathways, a reference agonist, and the same receptor/cell system; descriptive pathway preference is used when those requirements are not met. “A, FDA-approved for obesity; I, investigational (phase shown in Table 3). * Approved for specific genetic and hypothalamic obesity indications”.

### 5.2. Peripherally Acting Agents

Orlistat, approved in 1999 and available over the counter since 2007, is a non-systemic gastrointestinal lipase inhibitor. It irreversibly inhibits gastric and pancreatic lipases, preventing the hydrolysis of dietary triglycerides into absorbable free fatty acids and monoglycerides [15,90,91]. This mechanism reduces fat absorption by approximately 30%, leading to a caloric deficit without affecting appetite. Orlistat is most effective in individuals consuming high-fat diets. The XENDOS trial, a four-year randomized study, demonstrated a placebo-adjusted weight loss of 2.4% and a 37.3% relative reduction in progression to type 2 diabetes over four years (cumulative incidence 6.2% versus 9.0%); this is a whole-cohort figure that was driven by participants with impaired glucose tolerance at baseline, with no significant effect in those with normal glucose tolerance [59]. Additional benefits include modest improvements in lipid profiles and waist circumference. However, its tolerability is limited by gastrointestinal side effects such as oily stools, fecal urgency, and steatorrhea, which occur in up to 91% of users and lead to discontinuation in 8–10% of cases [60]. While some studies suggest a potential role in reducing colorectal adenoma risk, others raise concerns about increased preneoplastic markers in animal models, warranting further investigation [93].

Gelesis100 (Plenity) is a non-systemic oral hydrogel medical device, authorized through the FDA De Novo pathway in 2019, rather than a pharmacologic anti-obesity medication. It is taken before meals and absorbs water in the stomach to form a three-dimensional matrix that promotes fullness by increasing gastric volume [83]. In a randomized controlled trial involving 436 adults, Gelesis100 resulted in 6.4% weight loss versus 4.4% with placebo, with 59% of participants achieving at least 5% weight loss and 27% achieving at least 10% [83]. It was well tolerated, with a safety profile comparable to placebo; its mechanism is mechanical and non-systemic rather than receptor mediated.

### 5.3. Centrally Acting Agents

Phentermine, the first of the modern FDA-approved appetite suppressants (1959; amphetamine-type agents had been marketed for weight loss from the 1930s, before efficacy standards were introduced in 1962), is a sympathomimetic amine that increases norepinephrine release in the hypothalamus, thereby suppressing appetite [55,56]. Though approved only for short-term use (up to 12 weeks), it remains widely prescribed due to its affordability and efficacy. In clinical trials, phentermine has shown significant short-term weight loss compared to placebo, with dry mouth and insomnia being the most common side effects. It is contraindicated in individuals with cardiovascular disease, uncontrolled hypertension, hyperthyroidism, glaucoma, or recent monoamine oxidase (MAO) inhibitor use [8,57].

Topiramate, originally approved for epilepsy and migraine prophylaxis, contributes to weight loss through multiple mechanisms: enhancing GABA-mediated activity, inhibiting carbonic anhydrase, and modulating glutamate and neuropeptide Y pathways [90]. It may also increase thermogenesis and act as a neurostabilizer. However, cognitive side effects such as memory impairment and attention deficits have been reported, particularly at higher doses.

The combination of phentermine and topiramate extended release (PHEN/TPM ER) was approved in 2012 for long-term obesity management. This complementary formulation enhances appetite suppression and energy expenditure. Phase III trials EQUIP, CONQUER, and SEQUEL demonstrated significant, dose-dependent weight loss and improvements in cardiometabolic parameters. For example, in the CONQUER trial, participants lost 8.1–10.2 kg over 56 weeks compared to 1.4 kg with placebo [64]. The SEQUEL extension confirmed sustained weight loss over two years and additional benefits in blood pressure, lipid profile, and obstructive sleep apnea [61]. Common side effects include paresthesia, dry mouth, constipation, and insomnia. Due to teratogenic risks, the FDA mandates a Risk Evaluation and Mitigation Strategy (REMS) to inform prescribers and women of childbearing potential about the increased risk of orofacial clefts with first-trimester exposure to topiramate [94].

Naltrexone/bupropion (NB) is a centrally acting combination therapy approved in 2014. Bupropion, a dopamine and norepinephrine reuptake inhibitor, activates POMC neurons in the hypothalamus, leading to the release of α-melanocyte-stimulating hormone and β-endorphin. Naltrexone, an opioid receptor antagonist, blocks the autoinhibitory feedback of β-endorphin on POMC neurons, thereby sustaining appetite suppression [44]. This dual mechanism also modulates reward pathways, enhancing self-control and reducing hedonic eating [95]. The COR-I, COR-II, COR-BMOD, and COR-Diabetes trials demonstrated weight reductions of 6.1–11.5% with NB versus 1.2–5.1% with placebo [65,66,67,96]. In patients with T2DM, NB also improved Glycated Hemoglobin (HbA1c), triglycerides, and high-density lipoprotein cholesterol levels. However, gastrointestinal side effects (nausea, constipation, vomiting) and central nervous system effects (headache, dizziness) are common. The combination is contraindicated with MAO inhibitors due to the risk of hypertensive crisis [97,98].

## 6. Repurposed Antidiabetic Drugs in Obesity Pharmacotherapy

Several antidiabetic medications have demonstrated weight-reducing properties and are increasingly being repurposed for obesity management. These agents, originally developed for glycemic control in T2DM, exert their effects through diverse pharmacodynamic mechanisms that also influence energy balance, appetite regulation, and adiposity.

GIP receptor modulation represents a nuanced pharmacodynamic paradigm in weight regulation, as both receptor agonism and antagonism have been associated with weight loss under different therapeutic contexts [99]. GIP receptor agonism, particularly when combined with GLP-1 receptor activation, appears to enhance insulin sensitivity and modulate central appetite pathways, thereby supporting weight reduction [100]. Conversely, GIP receptor antagonism has been shown to limit adipose-tissue lipid storage and attenuate anabolic signaling, promoting reduced fat accumulation [101]. Recent preclinical evidence indicates that long-acting GIPR agonism and GIPR antagonism can reduce body weight through distinct mechanisms: agonism engaged GABAergic GIPR-positive circuits and reduced intake independently of GLP-1R, whereas antagonism depended on intact GLP-1R signaling and produced dorsal-vagal-complex transcriptional changes resembling GLP-1R activation [101].

Metformin, a biguanide widely used in the management of type 2 diabetes mellitus (T2DM), has shown modest weight-loss effects, typically ranging from 1 to 3 kg [102,103]. Its mechanisms include activation of AMP-activated protein kinase (AMPK), reduction in hepatic gluconeogenesis, and enhancement of insulin sensitivity. Metformin may also suppress appetite through central hypothalamic pathways and modulation of the gut microbiota [104]. Although not FDA-approved for obesity, it is frequently used off-label, particularly in patients with insulin resistance or polycystic ovary syndrome [105].

Sodium–glucose cotransporter 2 (SGLT2) inhibitors, such as canagliflozin and dapagliflozin, promote urinary glucose excretion, leading to net caloric loss [106,107]. In people with T2DM, this mechanism generally produces modest weight reduction together with osmotic diuresis and natriuresis. SGLT2 inhibitors are not approved specifically for obesity; their weight effect is best interpreted as a secondary metabolic consequence in patients treated for diabetes or cardiorenal indications rather than as established stand-alone obesity pharmacotherapy [106,107].

Other antidiabetic agents, such as DPP-4 inhibitors, have minimal impact on body weight and are generally weight-neutral. However, their combination with GLP-1 receptor agonists or SGLT2 inhibitors may offer additive benefits in select patient populations [108].

The repurposing of antidiabetic drugs underscores the importance of understanding pharmacodynamic mechanisms that extend beyond glycemic control. These agents offer additional tools for managing obesity, particularly in patients with coexisting metabolic disorders, and highlight the potential for dual-purpose pharmacotherapies in clinical practice.

## 7. GLP-1 Receptor Agonists and the Pharmacodynamic Basis of Incretin and Glucagon Pathways

For a concise primer on GLP-1, GIP, and glucagon pathways, see Section 3; subsequent sections refer to those mechanisms only where they inform pharmacodynamic clinical translation. This section focuses on how GLP-1-based therapies translate receptor activation into clinical efficacy, safety, and dose–response behavior rather than re-describing foundational physiology [30,33,34,35,84].

At the receptor level, liraglutide and semaglutide preserve high-potency human GLP-1R activation while fatty-acid acylation increases albumin binding and extends exposure. In human GLP-1R expression systems, their cAMP potency lies in the low-picomolar range, whereas semaglutide binding affinity is lower than liraglutide because structural changes were optimized for albumin binding and duration rather than maximal receptor affinity alone. Ecnoglutide illustrates a different strategy: cAMP EC_50_ is 0.018 nM, while measurable receptor internalization requires concentrations above 10 micromolar, indicating pronounced cAMP pathway preference [53,84,86,87].

These molecular differences must be interpreted together with exposure. Sustained free-drug concentrations, receptor residence, and repeated access to gut–brain and hypothalamic GLP-1R determine clinical efficacy more directly than a single in vitro EC_50_. The gradual titration used for liraglutide and semaglutide is therefore a pharmacodynamic strategy: it progressively increases receptor engagement while allowing adaptation to nausea, vomiting, and delayed gastric emptying, which are predominantly on-target GLP-1R effects [85,109].

Liraglutide, a long-acting GLP-1 receptor agonist (GLP-1RA), was initially approved in 2010 for T2DM at a dose of 1.8 mg/day and later at 3.0 mg/day for chronic weight management [84,110]. Its pharmacodynamic effects include glucose-dependent insulin secretion, inhibition of glucagon secretion, delayed gastric emptying, and appetite suppression [84,85,110]. Acylation and albumin binding confer a plasma half-life of approximately 13 h, supporting once-daily administration [84,110]. In the SCALE Obesity and Prediabetes trial, 63.2% of participants receiving liraglutide achieved at least 5% weight loss and 33.1% achieved more than 10% weight loss at 56 weeks [69]. Gastrointestinal adverse effects, including nausea, vomiting, diarrhea, and dyspepsia, are common and may lead to discontinuation, although they often improve with continued treatment and dose titration [14,85].

Semaglutide, a once-weekly GLP-1RA, offers enhanced efficacy and a longer duration of action. Approved in 2021 for chronic weight management, it has demonstrated superior weight loss compared to liraglutide and placebo [12,111]. In the STEP 1–5 trials, semaglutide 2.4 mg weekly led to 12–15% average weight loss, with up to 86% of participants achieving ≥ 5% weight reduction [6,45]. The STEP 8 trial further confirmed its superiority over liraglutide, with semaglutide achieving a mean body-weight reduction of 15.8% versus 6.4% with liraglutide at week 68 (these are body-weight, not fat-mass or adiposity, values) [111]. Semaglutide also improves glycemic control, reduces appetite, and alters food preferences [112]. Oral semaglutide formulations have shown promise as well. In the OASIS 1 trial, oral semaglutide 50 mg achieved a 15.1% body mass reduction in non-diabetic adults [113], while the PIONEER 1 trial demonstrated significant HbA1c reduction and weight loss in T2DM patients, supporting its dual role in glycemic and weight control [114].

Orforglipron (Foundayo), a once-daily oral nonpeptide GLP-1 receptor partial agonist, received FDA approval on 1 April 2026 for chronic weight management in adults with obesity or adults with overweight and at least one weight-related comorbidity. In the Phase III ATTAIN-1 trial (*n* = 3127; 72 weeks; adults with obesity, or overweight with at least one weight-related complication, and without diabetes), mean body-weight reductions were 7.5% (6 mg), 8.4% (12 mg) and 11.2% (36 mg) versus 2.1% with placebo under the treatment-regimen estimand, and 7.8%, 9.3% and 12.4% versus 0.9% with placebo under the efficacy estimand [10]. Because the two estimands differ by roughly one percentage point at the lowest dose and by more than three percentage points in the placebo arm, the estimand is stated explicitly here and wherever these values are reproduced in Table 1. At 36 mg, 71.8% of participants achieved at least 5% and 54.6% at least 10% weight loss (treatment-regimen estimand), accompanied by reductions in waist circumference (−10.1 cm), systolic blood pressure (−6.3 mmHg) and triglycerides (−20.2%). Body weight had not plateaued at week 72, so the reported magnitude should not be interpreted as a maximal effect, and no hepatic safety signal was observed, in contrast to the earlier nonpeptide agonists danuglipron and lotiglipron [10]. Its small-molecule scaffold permits oral administration without an empty-stomach requirement, while gastrointestinal adverse effects and gradual dose escalation remain consistent with on-target GLP-1R pharmacology [10,77,78,79]. At the receptor level, orforglipron binds the human GLP-1R with a Ki of approximately 1 nM and behaves as a partial, cAMP-preferring agonist: it activates Gs-cAMP signaling while recruiting little beta-arrestin and producing limited receptor internalization [78]. Its apparent efficacy is receptor-density dependent, approaching full agonism where receptor reserve is high and partial agonism where receptor expression is limiting, so “partial agonist” should be read as a statement of intrinsic efficacy rather than of the maximal attainable clinical response. As for the other agents discussed here, no formal operational-model bias factor has been reported, and this profile is therefore described as pathway preference rather than quantified bias [52,78]. Structurally, orforglipron occupies a nonpeptide pocket in the upper helical bundle formed by the extracellular domain; extracellular loop 2; and transmembrane helices 1, 2, 3 and 7, and its activity depends on the primate-specific residue Trp33 of the extracellular domain; rodents carry serine at this position and do not respond, so wild-type rodent efficacy data do not translate for this class and humanized or Glp1r S33W models are required [78,115]. Pharmacokinetically, the small-molecule scaffold confers an elimination half-life of approximately 29–49 h with predominantly CYP3A4-mediated metabolism, and, unlike oral semaglutide, requires neither the absorption enhancer sodium N-(8-[2-hydroxybenzoyl]amino)caprylate nor fasted administration the pharmacokinetic basis for the dosing convenience noted above [116].

The next generation of anti-obesity agents builds on GLP-1–based mechanisms but extends them through dual and triple receptor targeting, as covered in Section 8 and Section 9. In this section, we note only that multi-agonists combine complementary pathways to enhance appetite suppression, improve metabolic efficiency, and reduce compensatory mechanisms. Detailed mechanisms and pharmacodynamic profiles are provided in the dedicated multi-agonist sections that follow [34].

## 8. Dual GLP-1 and GIP Receptor Agonists (GIPRAs): Clinical Development and Translational Insights

Early clinical trials with dual agonists such as NNC0090-2746 and RG7697 demonstrated modest weight loss and glycemic improvements in patients with type 2 diabetes who were inadequately controlled on metformin [35]. However, development of RG7697 was discontinued because of higher discontinuation rates and the need for daily injections, which limited its clinical utility compared with once-weekly GLP-1 receptor agonists.

Combination approaches have also been explored preclinically; for example, liraglutide was studied with phentermine in mice, but such findings should not be extrapolated to long-term human efficacy without clinical confirmation [117]. Tirzepatide represents a distinct unimolecular strategy: it combines GIPR and GLP-1R agonism within a single molecule rather than simply co-administering two appetite-suppressive drugs [34,35,54]. Its receptor pharmacology is detailed in Section 9.1.

## 9. Multi-Receptor Agonism: A New Frontier in Obesity Pharmacotherapy

Recent anti-obesity drug development intentionally manipulates receptor potency ratios and polypharmacology rather than assuming equal activity at every target. Tirzepatide is imbalanced toward GIPR engagement and displays cAMP-preferring partial agonism at GLP-1R, whereas retatrutide is a calibrated triple agonist with its highest potency at GIPR; its GLP-1R-versus-GCGR ordering differs between cAMP and binding assays run in separate receptor systems, so cross-receptor ranking is approximate. These profiles distribute pharmacologic activity across complementary pathways without requiring equipotent receptor activation [26,54,75]. The following subsections relate these receptor-level properties to dose–response, efficacy, and adverse effects.

The shift from single-target therapies to multi-receptor incretin-based agonists represents a major evolution in obesity pharmacotherapy. By engaging GLP-1, GIP, and glucagon receptors in calibrated combinations, these agents may address some physiological adaptations that limit the long-term effects of isolated GLP-1 receptor agonism [34,35]. Clinical findings with tirzepatide and Phase II and III data for retatrutide demonstrate the potential for deeper weight-loss responses, but long-term safety, cardiovascular effects, and real-world durability require continued evaluation [80,90,118].

### 9.1. Dual GLP-1 and GIP Receptor Agonists

Tirzepatide is pharmacologically imbalanced toward GIPR engagement rather than a balanced or GLP-1R-dominant dual agonist. In low-density human receptor systems, it is fully efficacious at GIPR (Gs EC_50_ 0.379 nM) but a partial agonist at GLP-1R (51% Emax; Gs EC_50_ 0.617 nM). At GIPR, its beta-arrestin recruitment resembles native GIP; at GLP-1R, beta-arrestin efficacy is below 10% and receptor internalization is reduced to approximately 43.6% of the GLP-1 response. Thus, the documented cAMP preference occurs at GLP-1R, while GIPR signaling remains broadly similar to native GIP [54].

This receptor profile helps explain, but does not by itself prove, the clinical magnitude of weight loss. Stronger GIPR occupancy adds insulinotropic, adipose-tissue, and central effects, while partial and cAMP-preferring GLP-1R signaling may preserve surface receptor availability and sustained cellular responsiveness. Clinical dose escalation still remains constrained by GLP-1R-mediated gastrointestinal effects, and efficacy also depends on the five-day half-life and continuous dual-receptor exposure [54,76].

Tirzepatide is the most clinically advanced dual GLP-1/GIP receptor agonist and has demonstrated robust effects on both weight and glycemic outcomes. In a 26-week Phase II study of 318 adults, tirzepatide produced dose-dependent weight reductions of −0.9, −4.8, −8.7, and −11.3 kg at 1, 5, 10, and 15 mg, respectively; the 11.3 kg figure is the highest dose, not a mean across doses, compared with 2.7 kg for dulaglutide 1.5 mg and 0.4 kg for placebo [42]. Across doses of 5–15 mg, weight loss in SURMOUNT-1 ranged from 15.0% to 20.9% under the treatment-regimen estimand (placebo −3.1%) and from 16.0% to 22.5% under the efficacy estimand (placebo −2.4%); the two estimands should be reported separately rather than combined into a single range, with gastrointestinal events (nausea, vomiting, diarrhea, reduced appetite) occurring in a minority of participants and generally remaining mild to moderate [76]. Phase III data reinforce these findings: SURPASS trials confirm greater HbA1c and weight reductions than semaglutide, although the comparator in SURPASS-2 was semaglutide 1 mg rather than the 2.4 mg dose used for weight management; in the dedicated obesity comparison SURMOUNT-5, tirzepatide produced -20.2% versus −13.7% with semaglutide 2.4 mg at 72 weeks [119], while the SURMOUNT studies document 12.8–20.9% weight loss (SURMOUNT-2, −12.8% to −14.7% in type 2 diabetes; SURMOUNT-4, −20.9% during the open-label lead-in; and SURMOUNT-3, a further ~21% after a lifestyle lead-in this range therefore spans different trials, populations and estimands rather than a single dose–response) depending on population and dose [118,120,121]. SURMOUNT-4 further showed sustained reductions through 52 weeks but partial regain after treatment withdrawal, indicating the need for continued therapy to maintain outcomes [75]. Additional improvements in cardiometabolic markers, including waist circumference, blood pressure, and lipid levels, have been consistently observed. Although gastrointestinal effects remain the most common adverse events, tirzepatide’s overall safety profile is acceptable, and longer-term cardiovascular outcomes are under active investigation [76].

### 9.2. Dual GLP-1 and Glucagon Receptor Agonists

Dual GLP-1R/GCGR agonists require a narrow pharmacodynamic balance. In CHO-K1 cells expressing human receptors, survodutide produced cAMP EC_50_ values of 0.33 nM at GLP-1R and 0.52 nM at GCGR. GLP-1R activation suppresses appetite and supports glucose-dependent insulin secretion, whereas hepatic GCGR activation promotes substrate mobilization and energy expenditure but can increase heart rate or oppose glucose lowering if excessive. The therapeutic profile therefore depends on the relative exposure and efficacy at both receptors, not simply on the presence of dual activity [88,122,123]. Interpretation requires the native ligands assayed in the same system (GLP-1 approximately 0.06 nM; glucagon approximately 0.02 nM): survodutide is about 5-fold less potent than GLP-1 at GLP-1R but about 26-fold less potent than glucagon at GCGR. In absolute terms, the molecule is therefore near-balanced with a slight GLP-1R lean, whereas relative to the endogenous hormones it is GLP-1R-weighted rather than glucagon-dominant, which frames the glucagon arm as a modulatory addition to a GLP-1R-driven anorectic effect rather than as the principal driver [88]. Consistent with this, the acute suppression of food intake by survodutide is preserved in wild-type but lost in GLP-1R-knockout mice, while glucagon-receptor engagement in vivo is evidenced by increased hepatic NNMT expression and circulating FGF-21 [88]. No beta-arrestin, internalization or bias data have been published for survodutide, so no signaling-bias claim is made for this agent.

Survodutide demonstrated dose-dependent weight reduction in Phase II studies. In the Phase III SYNCHRONIZE-1 trial (*n* = 725; 76 weeks), mean body-weight change at week 76 was −12.2% with 3.6 mg and −13.0% with 6.0 mg versus −5.4% with placebo under the treatment-regimen estimand and −15.3% and −16.6% versus −3.2% under the efficacy estimand [124]. The unusually large placebo response reflects the use of prohibited GLP-1-based medication by more than 16% of placebo-assigned participants during the trial rather than an intensive lifestyle co-intervention, and it accounts for most of the divergence between the two estimands; cross-trial comparison with earlier programs, whose placebo arms had little such crossover, is correspondingly limited. Gastrointestinal events occurred in 81% (3.6 mg) and 90% (6.0 mg) versus 48% with placebo and led to discontinuation in 17.8% and 20.2% versus 2.9%, and resting heart rate rose by approximately 3.2–3.5 bpm, consistent with the glucagon-receptor component and intermediate between pure GLP-1 receptor agonists and the triple agonist retatrutide. In a magnetic-resonance substudy, liver fat fell by 63.1% and visceral fat by 34.0% at 6.0 mg, reductions disproportionate to total weight loss and consistent with hepatic GCGR engagement [124]. Mazdutide has published Phase III GLORY-1 efficacy data and received approval in China for chronic weight management, while pemvidutide has completed the Phase II MOMENTUM obesity trial [125,126]. These findings support calibrated dual incretin–glucagon agonism as a strategy to enhance energy expenditure while preserving the appetite-suppressive effects of GLP-1 receptor activation. This strategy has not, however, yet produced greater weight loss than best-in-class single or dual incretin agents: survodutide’s efficacy-estimand weight loss is comparable to that reported for semaglutide 2.4 mg and lower than that reported for tirzepatide, albeit across non-comparable trials differing in population, duration, diet co-intervention and estimand. On current evidence the distinctive contribution of the glucagon-receptor component is hepatic and visceral fat mobilization, supported by the Phase II MASH data and by the imaging findings above, rather than incremental total weight loss, and it is accompanied by an additional heart-rate signal and a substantially higher rate of gastrointestinal discontinuation [46,88,123,124].

### 9.3. Triple Agonists: GLP-1, GIP, and Glucagon

Retatrutide is not equipotent across its three receptors. In human receptor assays, cAMP EC_50_ values were 0.0643 nM at GIPR, 0.775 nM at GLP-1R, and 5.79 nM at GCGR, with corresponding binding Ki values of 0.057, 7.2, and 5.6 nM. This potency hierarchy provides strong GIPR activity, clinically meaningful GLP-1R satiety signaling, and a lower-potency glucagon component that adds hepatic energy expenditure as exposure rises. The dose-dependent efficacy and transient heart-rate increase observed clinically are therefore consistent with graded multi-receptor engagement rather than uniformly balanced triagonism [26,80].

Retatrutide is an investigational triple agonist targeting GLP-1R, GIPR, and GCGR, designed to combine appetite suppression, insulinotropic effects, and glucagon-mediated metabolic activity within a single pharmacologic profile [26,80]. In a 48-week Phase II study, retatrutide produced dose-dependent mean weight reductions of 8.7%, 17.1%, 22.8% and 24.2% at 1, 4, 8 and 12 mg respectively, versus 2.1% with placebo (efficacy estimand), with gastrointestinal adverse events and transient heart-rate increases among the principal tolerability findings [80]. The receptor profile is calibrated rather than equipotent, with greater GIPR potency than GLP-1R or GCGR potency [26]. Phase III trials are needed to define long-term safety, cardiovascular outcomes, durability, and the clinical contribution of each receptor component. Since this manuscript was prepared, the Phase III TRIUMPH program has reported the following: in TRIUMPH-1, mean weight reductions were 19.0%, 25.9% and 28.3% with 4, 9 and 12 mg versus 2.2% with placebo at 80 weeks (efficacy estimand), with further readouts in TRIUMPH-4 and TRANSCEND-T2D-1 [127].

### 9.4. Mechanistic Advantages and Clinical Implications

Multi-receptor agonists can exceed single-pathway efficacy when receptor contributions are complementary and appropriately weighted. GLP-1R activity suppresses appetite and slows gastric emptying, GIPR activity supports glucose-dependent insulin secretion and adipose-tissue physiology, and GCGR activity can increase hepatic substrate turnover and energy expenditure. The benefit therefore arises from the integrated exposure–response profile across receptors, not from an assumption that all receptors should be activated equally [26,34,35,54,88].

Multi-receptor design may also reduce reliance on maximal stimulation of a single receptor, but evidence that it prevents receptor desensitization or rebound appetite in humans remains incomplete. The more defensible interpretation is that complementary receptor engagement broadens the physiological response while preserving dose-limiting tolerability. Claims of synergy, reduced desensitization, or formal signaling bias should therefore be supported by matched receptor/pathway assays rather than inferred from clinical weight loss alone [52,54].

Dose–response across multi-agonists reflects both receptor potency ratios and pharmacokinetic exposure. Tirzepatide reaches clinically relevant GIPR and GLP-1R engagement throughout weekly dosing, whereas increasing retatrutide exposure progressively recruits the lower-potency GCGR component. This framework links receptor pharmacology to greater weight loss while also anticipating gastrointestinal symptoms from GLP-1R and heart-rate effects from GCGR activation [26,54,75,80].

## 10. Pharmacodynamic Precision and Personalized Treatment Selection

Precision treatment is most established when a defined molecular or pathway defect predicts response to a receptor-targeted drug. Setmelanotide and metreleptin, whose use depends on genotype- or pathway-defined eligibility, illustrate this principle (Section 13) [30,47,71,89,128].

For common obesity, pharmacodynamic personalization is currently based more on clinical phenotype and treatment tolerance than on validated genomic algorithms. Examples include prioritizing incretin-containing regimens when type 2 diabetes or marked appetite dysregulation is present, considering GLP-1R/GCGR agents when hepatic steatosis and energy-expenditure targets are relevant, and considering body-composition-directed approaches such as ActRII blockade when the preservation of lean mass is a major objective. These are mechanism-informed choices, but comparative biomarkers that prospectively select one receptor profile over another still require validation [49,122,123].

Dose titration is another practical form of personalization. Escalation can be guided by weight response, glycemic benefit, gastrointestinal tolerability, heart-rate changes, and persistence of appetite suppression, thereby matching exposure to the individual therapeutic window. Pharmacogenomic, metabolomic, and digital prediction tools remain promising research strategies, but they should not be presented as established precision-medicine standards until prospectively validated [7,129].

Functional genomics and CRISPR-based target-validation studies may refine future pathway eligibility, but they remain research tools rather than established treatment-selection criteria [130].

## 11. Anti-Obesity Drugs Under Development: A Pharmacodynamics Perspective

While early experimental approaches, such as obesity vaccines, have not demonstrated meaningful efficacy in humans and have faced safety concerns in preliminary trials [131], current development efforts focus on pharmacodynamically rational strategies with clearer translational potential. Next-generation agents target complementary central and peripheral pathways to address the homeostatic mechanisms that limit sustained responses to single-pathway therapies. These candidates include multi-receptor incretin–glucagon agonists, amylin-based co-agonists, GDF15-pathway agents, ActRII inhibitors, and peripherally restricted CB1R modulators, each with distinct potential advantages and mechanism-linked limitations.

Emerging anti-obesity pharmacotherapies use several distinct pharmacodynamic strategies. Multi-receptor incretin–glucagon agonists combine appetite, insulinotropic, and energy-mobilization pathways [26,80,88,132]. Amylin analogs and amylin–incretin combinations enhance satiation and slow gastric emptying [11,133,134,135]. MC4R agonism can restore melanocortin signaling in selected pathway-defined disorders [30,47,70]. GDF15 analogs act through the GFRAL/RET complex in the hindbrain to reduce food intake, although nausea and emesis can limit exposure [50,51]. These platforms illustrate how pharmacodynamic innovation may deepen efficacy while creating new requirements for receptor balance, assay-context interpretation, and tolerability management.

Table 3 provides an overview of selected emerging, investigational, repurposed, and regionally approved anti-obesity agents, summarizing their primary receptor targets, pharmacodynamic mechanisms, efficacy signals, and stage of clinical development or regional regulatory status.

**Table 3:** Selected emerging, investigational, repurposed, and regionally approved anti-obesity agents: mechanisms, pharmacodynamic profiles, clinical progress, and efficacy outcomes.

Drug Candidate/Reference(s)/Clinical-Trial Registration	Mechanism of Action and Pharmacodynamic Effects	Pharmacodynamic and Clinical Interpretation	Clinical Development Phase/Status	Reported Clinical Efficacy (Weight Loss)	Common Adverse Effects
Cagrilintide [133] (Phase II: NCT03856047; Phase III RENEW 1: NCT07220642; RENEW 2: NCT07220759)	Amylin receptor agonist Reduced appetite (central satiety signaling) Delayed gastric emptying	Adds sustained satiation and meal size reduction via long-acting amylin-receptor activation.	I; Phase III program ongoing; published Phase II data available.	6.0% to 10.8% from baseline to week 26.	Nausea, administration-site reactions
CagriSema [134,135] (NCT05567796)	Dual GLP-1R and amylin-receptor agonism Enhances satiation and reduces appetite Slows gastric emptying Supports glucose-dependent insulin secretion	Amplifies weight loss by combining GLP-1 and amylin-receptor satiety pathways.	I; Phase III	20.4% mean weight reduction at week 68 (vs. 3.0% with placebo). (treatment-policy estimand; 22.7% with full adherence. A New Drug Application was filed in December 2025 and the combination is not yet approved.)	Mainly transient, mild-to-moderate gastrointestinal events, including nausea, vomiting, diarrhea, constipation, and abdominal pain.
MariTide (AMG 133) [92,136] (NCT05669599; Phase III: NCT06858839)	GLP-1R agonist/GIPR antagonist Appetite suppression Potential modulation of energy expenditure	Improves weight loss by combining GIPR antagonism with GLP-1 driven satiety.	I; Phase III ongoing	Phase II: 12.3–16.2% mean weight reduction at week 52 (participants without diabetes; treatment-policy estimand).	Nausea and vomiting; dose escalation improved tolerability.
EMP-16 [48,137] (Published Phase II trials; EudraCT 2022-003320-40; POEM tolerability pilot: NCT06993428)	Dual alpha-glucosidase and pancreatic-lipase inhibition: reduced carbohydrate digestion and reduced dietary-fat hydrolysis.	Reduces carbohydrate and fat absorption; gastrointestinal adverse effects reflect local enzyme inhibition.	I; Phase II; POEM tolerability pilot in preparation for Phase III.	Initial 26-week trial: −5.53% and −6.25% vs. −0.83% with placebo. Later 6-month trial: −7.73% vs. −5.78% with modified-release orlistat and −5.13% with conventional orlistat.	Predominantly gastrointestinal; no serious adverse events occurred in the later Phase II trial. The separate POEM pilot evaluates gastrointestinal tolerability during dose escalation.
Bimagrumab [49] (NCT03005288)	ActRII inhibitor Blocks myostatin pathway, leading to increased muscle mass Promotes fat mass reduction	Promotes lean-mass preservation by blocking activin signaling and increasing skeletal-muscle mass.	I; Phase II	At week 48: body weight −6.5%, fat mass −20.5%, and lean mass + 3.6% from baseline.	Diarrhea, muscle cramps
LY3463251 [50] (NCT03764774)	Long-acting GDF15 analog and GFRAL/RET receptor agonist: activates brainstem anorectic signaling and reduces food intake.	Provides proof of mechanism for GDF15-GFRAL/RET appetite regulation; human weight loss was modest and nausea/emesis were dose related.	I; Phase I	~3.0% weight loss from baseline to week 12	Nausea, vomiting
INV-202 [37] (Phase Ib: NCT05282446; Phase II: NCT05891834)	Peripherally restricted CB1R inverse agonist Modulates peripheral appetite and metabolic signaling Improves adipose and hepatic metabolic measures	Reduces appetite and weight through peripheral CB1-receptor blockade.	I; Phase Ib completed; Phase II completed.	Phase Ib: ~3.3% weight loss (vs. 0.5% weight gain with placebo) after 28 days.	Nausea, decreased appetite, and diarrhea; no serious adverse events were reported in the Phase Ib study.
Carbetocin [138,139] (CARE-PWS: NCT03649477; COMPASS PWS: NCT06173531)	Oxytocin receptor agonist Suppresses appetite and reduces food intake	Investigated for hyperphagia and behavioral symptoms in Prader–Willi syndrome; not a general anti-obesity therapy.	I; Phase III	CARE-PWS showed nominal benefit with 3.2 mg three times daily; the confirmatory COMPASS PWS trial did not demonstrate efficacy versus placebo for hyperphagia.	Headache and pyrexia were the most frequent events in COMPASS PWS; earlier reports included flushing and nasopharyngitis.
Metformin [140]	AMPK pathway activator Decreased hepatic glucose output Increased peripheral glucose uptake and reduced appetite	Supports weight loss and glycemic control through improved insulin sensitivity and reduced hepatic glucose output.	Rp; approved for T2DM, not obesity.	Meta-analysis: modest BMI reduction (WMD −0.98 kg/m^2^; 95% CI, −1.25 to −0.72).	Diarrhea, nausea/vomiting, flatulence, indigestion, and abdominal discomfort; hypoglycemia is uncommon with monotherapy.
Dapagliflozin [141] (NCT02338193)	SGLT2 inhibitor Increased urinary excretion of glucose	Promotes weight loss through urinary glucose excretion via SGLT2 inhibition	Rp; approved for T2DM/cardiorenal indications, not obesity.	In the cited 24-week study, dapagliflozin–metformin produced greater weight reduction than metformin monotherapy in women with overweight/obesity after recent gestational diabetes.	Genitourinary infections, dehydration
Exenatide [142] (NCT00500370)	GLP-1 receptor agonist Enhances insulin release, suppresses glucagon Delays gastric emptying and suppresses appetite	Supports weight loss through GLP-1 mediated satiety and slowed gastric emptying.	Rp; approved for T2DM, not obesity	−5.1 kg from baseline; placebo-subtracted difference −3.3% at week 24.	Nausea, diarrhea
Ecnoglutide (XW003) [143,144] (NCT05813795)	GLP-1 receptor agonist Suppresses appetite Delays gastric emptying Enhances glucose-dependent insulin secretion	Supports weight loss through long-acting GLP-1 mediated satiety.	R (China, 2026); Phase III	Mean weight change at week 40: −9.1%, −10.9%, and −13.2% with 1.2, 1.8, and 2.4 mg, respectively, vs. 0.1% with placebo.	Mostly mild-to-moderate gastrointestinal events, including decreased appetite, diarrhea, nausea, and vomiting.
TG103 [145] (Phase Ib: NCT04855292; Phase II: NCT05299697; Phase III: NCT05997576)	GLP-1 receptor agonist Suppresses appetite Delays gastric emptying	Drives weight loss and metabolic improvement through long-acting GLP-1 receptor activation.	Phase III program; published Phase Ib evidence available.	>5% mean weight loss at week 12 in the published Phase Ib study.	Decreased appetite, nausea, diarrhea, injection-site reactions, and vomiting; most events were grade 1–2, with no serious adverse events.
MDR-001 [146] (NCT06606483)	Oral GLP-1 receptor agonist Suppresses appetite Delays gastric emptying Improves waist circumference and lipid measures in company-reported results	Company-reported Phase IIb topline results support oral GLP-1R agonism; the cited evidence is a company press release rather than a peer-reviewed clinical publication.	Phase IIb; company release	Company-reported: 8.2–10.3% mean weight reduction at week 24 (2.5% with placebo).	Company-reported: no treatment-related serious adverse events; most events were mild-to-moderate gastrointestinal symptoms.
Beinaglutide [147] (ChiCTR1900023428)	GLP-1 receptor agonist Suppresses appetite Delays gastric emptying	Supports weight loss through short-acting GLP-1-mediated satiety.	Rp; Phase III	Mean body-weight change at week 16: −6.0% vs. −2.4% with placebo.	Nausea, dizziness, vomiting
Survodutide [46,124] (NCT06066515)	GLP-1R and GCGR agonist Suppresses appetite Increases energy expenditure	Delivers weight-loss benefits through dual GLP-1 and glucagon-receptor activation	Phase III	Mean weight change at week 76: −12.2% (3.6 mg) and −13.0% (6.0 mg), vs. −5.4% with placebo. (treatment-regimen estimand; −15.3% and −16.6% vs. −3.2% under the efficacy estimand).	Gastrointestinal symptoms, typically mild to moderate (80.9–89.7% vs. 47.9% with placebo).
Retatrutide (LY3437943) [26,76,77,80,81] ([TRIUMPH-1 registration number])	Triagonist targeting GIPR, GLP-1R, and GCGR Suppresses appetite Delays gastric emptying Increases hepatic substrate mobilization and energy expenditure	Calibrated, non-equipotent triple agonism; the glucagon component is recruited as exposure rises, consistent with dose-dependent efficacy and transient heart-rate increase	Phase III TRIUMPH-1 conference data, Phase II peer-reviewed	Mean weight change at week 80: −19.0% (4 mg), −25.9% (9 mg), and −28.3% (12 mg) vs. −2.2% with placebo (efficacy estimand; conference data). Phase II: −24.2% at 12 mg at 48 weeks.	Nausea, diarrhea, constipation, and vomiting; transient heart-rate increase.
Mazdutide [126,148] (GLORY-1: NCT05607680)	GLP-1R and GCGR agonist Suppresses appetite Increases energy expenditure	Delivers weight-loss and metabolic benefits through dual GLP-1 and glucagon-receptor activation.	R (China); Phase III elsewhere	Mean weight change at week 48: −11.00% with 4 mg and −14.01% with 6 mg, vs. 0.30% with placebo. (treatment-policy estimand; −12.05% and −14.84% vs. −0.47% under the efficacy estimand).	Mostly mild-to-moderate gastrointestinal adverse events.
Pemvidutide [125,149] (First-in-human Phase I: NCT04561245; MASLD Phase I: NCT05006885; Phase II MOMENTUM: NCT05295875)	GLP-1R and GCGR agonist Suppresses appetite Increases energy expenditure	Provides weight-loss benefits through dual GLP-1 and glucagon-receptor activation.	I; Phase II completed	At week 48: −10.3%, −11.2%, and −15.6% with 1.2, 1.8, and 2.4 mg, respectively, vs. −2.2% with placebo.	Mostly mild-to-moderate adverse events; one drug-related serious adverse event and minimal heart-rate increase in MOMENTUM.
Efocipegtrutide (HM15211) [150] (Phase I obesity: NCT03374241; Phase II NASH: NCT04505436)	Triagonist targeting GLP-1R, GIPR, and GCGR Promotes energy expenditure Lipid oxidation Enhanced satiety Decreases triglycerides	Triple GLP-1R/GIPR/GCGR agonism integrates satiety, insulinotropic activity, and glucagon-mediated energy expenditure.	I; Phase I obesity study completed; Phase II NASH study active, secondary review only.	No established long-term obesity efficacy from a primary obesity trial; the Phase I obesity study evaluated single-dose safety, pharmacokinetics, and pharmacodynamics.	Single-dose obesity study; no long-term obesity safety conclusion can be drawn.
SAR441255 [150,151] (NCT04521738)	Triagonist targeting GLP-1R, GIPR, and GCGR Lowers plasma glucose Reduces liver enzymes Lowers HbA1c and fasting plasma glucose	Provides weight-loss effects through triple GLP-1, GIP, and glucagon-receptor activation.	I; Phase I	No established human weight-loss efficacy; published human data are limited to single-dose safety/pharmacokinetics, while weight-loss efficacy was demonstrated preclinically.	Nausea, vomiting, dry mouth, and mouth ulceration; single doses were generally well tolerated.
NN1706 (NNC9204-1706; MAR423/RO6883746) [149,150] (NCT03095807; NCT03661879)	Fatty-acylated GLP-1R/GIPR/GCGR triagonist Balanced GLP-1R/GIPR activity with lower relative GCGR potency Integrates appetite, insulinotropic, and glucagon-mediated metabolic effects	Triple-receptor pharmacology produced clinically meaningful short-term weight loss, but cardiovascular tolerability signals limited further development.	I; Phase I	Up to 8.2% weight loss from baseline after 10 weeks.	Dose-related gastrointestinal events and persistent heart-rate increase; tachycardia/arrhythmia signals challenged further development.

R, regionally approved for obesity; Rp, repurposed; I, investigational. Evidence source is peer-reviewed publication unless otherwise indicated in the status column (conference, company release, or secondary review only).

Across investigational therapies, several pharmacodynamic patterns have emerged. Multi-receptor incretin–glucagon agonists produce large, dose-dependent reductions in energy intake and body weight [26,80,88]. Amylin–incretin combinations enhance satiation [11,133,134,135], whereas GDF15-pathway agents engage a separate hindbrain anorectic pathway [50,51]. ActRII blockade can increase lean mass while reducing fat mass [49], and peripherally restricted CB1R inverse agonism is intended to improve hepatic and adipose metabolic signaling while limiting central psychiatric exposure [37,38]. These mechanisms should not be treated as interchangeable, because efficacy and adverse effects depend on target distribution, exposure, and pathway-specific biology.

These approaches expand therapeutic diversity, but comparative evidence is still insufficient to conclude that any mechanism uniformly prevents weight-loss plateaus or improves long-term tolerability. Individual non-incretin agents are discussed in Section 14.

### 11.1. Single GLP-1 Receptor Agonists

Several emerging GLP-1R agonists have advanced through clinical development. Ecnoglutide, a long-acting GLP-1 analog that preferentially activates cAMP signaling over beta-arrestin recruitment, has completed Phase III evaluation. At week 40, mean body-weight changes were −9.1%, −10.9%, and −13.2% with 1.2, 1.8, and 2.4 mg, respectively, compared with 0.1% with placebo [53,109,144]. TG103, a GLP-1/Fc fusion protein, demonstrated at least 5% weight loss in early trials with extended exposure through Fc-mediated recycling and resistance to enzymatic degradation [145,152]. Company-reported Phase IIb topline results for MDR-001, an AI-designed oral GLP-1RA, indicated 8.2–10.3% weight reduction at 24 weeks with improvements in waist circumference, blood pressure, and lipid profiles; the cited evidence is a company press release rather than a peer-reviewed clinical publication [146]. Beinaglutide, approved in China for T2DM, produced −6.0% mean body-weight change at week 16 versus −2.4% with placebo in a Phase III obesity trial [147,153].

### 11.2. Dual GLP-1/Glucagon Receptor Agonists

Pemvidutide is a dual GLP-1R/GCGR agonist evaluated in both metabolic liver-disease and obesity programs. A separate 12-week Phase I MASLD study documented early liver-fat and body-weight effects [125]. In the 48-week Phase II MOMENTUM obesity trial, mean weight losses were 10.3%, 11.2%, and 15.6% with 1.2, 1.8, and 2.4 mg, respectively, compared with 2.2% with placebo; most adverse events were mild-to-moderate, with one drug-related serious adverse event and minimal heart-rate increase [154].

### 11.3. Triple Agonists (GLP-1/GIP/Glucagon)

Among multi-receptor strategies, triple agonists that simultaneously target GLP-1R, GIPR, and GCGR have entered human evaluation, but the maturity and direction of the evidence differ by candidate. Efocipegtrutide (HM15211) completed a single-dose Phase I study in participants with obesity, while its ongoing Phase II program is directed at biopsy-confirmed NASH rather than primary obesity efficacy [150]. SAR441255 established single-dose human safety and pharmacokinetics but has no established human weight-loss efficacy; its weight-loss evidence is preclinical [150,151]. NN1706 (NNC9204-1706) produced up to 8.2% weight loss after 10 weeks, but persistent heart-rate elevation and tachycardia/arrhythmia signals challenged further development [149]. These findings reinforce the need for candidate-specific interpretation of receptor balance, exposure–response, efficacy, and cardiovascular tolerability.

## 12. Medicinal Chemistry Considerations and Structure-Activity Relationships

The development of modern anti-obesity medications is a testament to the power of medicinal chemistry in optimizing drug efficacy, selectivity, and pharmacokinetic profiles. The evolution from early, non-specific agents to highly targeted multi-receptor agonists has been driven by a deep understanding of structure–activity relationships (SARs) [132]. Small-molecule agents: For centrally acting drugs like the phentermine/topiramate combination, the therapeutic effect arises from two distinct pharmacophores. Phentermine, a simple phenethylamine derivative, acts as a norepinephrine-releasing agent [155]. Topiramate, a sulfamate-substituted monosaccharide, has a more complex profile, interacting with GABA-A receptors and voltage-gated sodium channels [62,63]. The combination of naltrexone, an opioid antagonist with a morphinan scaffold, and bupropion, a cathinone derivative, targets the brain’s reward pathways, demonstrating how combining known pharmacophores can lead to complementary effects on appetite and craving [67]. Peptide-based therapeutics: The most significant recent advances have been in peptide engineering. Native GLP-1 has a very short half-life (1–2 min) due to rapid degradation by dipeptidyl peptidase-IV (DPP-4) and renal clearance. Medicinal chemists have overcome this challenge through several strategies [132]:

### 12.1. DPP-4 Resistance

The substitution of the alanine at position 8 with a non-natural amino acid like aminoisobutyric acid (Aib), as seen in semaglutide, sterically hinders the action of DPP-4.

### 12.2. Albumin Binding

Acylation with long-chain fatty acids allows the peptides to reversibly bind to serum albumin, creating a circulating depot and dramatically extending their half-life. Liraglutide is acylated with a C16 fatty acid, while semaglutide features a C18 diacid linker, further enhancing its binding affinity and duration of action to allow for once-weekly administration.

### 12.3. Multi-Receptor Agonism

Dual and triple agonists are single peptides engineered to produce intentional, and often unequal, potency at several receptors. Tirzepatide was built on a GIP-like backbone and retains stronger GIPR engagement while introducing partial, cAMP-preferring GLP-1R agonism. Retatrutide uses sequence substitutions, including Aib residues, together with lipidation to achieve a calibrated GIPR/GLP-1R/GCGR potency hierarchy. These examples show that receptor balance is created through sequence-dependent affinity, efficacy, signaling, and exposure rather than by equal activity at every receptor [26,54,132].

These examples highlight how rational drug design, focusing on SAR and pharmacokinetic optimization, has been pivotal in creating the highly effective anti-obesity therapies available today and in development [80].

## 13. Pharmacodynamic Precision in Genetic Obesity

Genetic obesity is included only as a focused example of receptor-pathway precision. Setmelanotide directly activates MC4R downstream of selected defects in POMC, PCSK1, LEPR, and Bardet–Biedl pathways, reducing hyperphagia when the melanocortin circuit remains pharmacologically accessible. Its high MC4R potency and weaker activity at MC1R/MC3R explain both pathway-specific efficacy and off-target hyperpigmentation; these properties do not support use in unselected common obesity. Its separate indication for acquired hypothalamic obesity is pathway-defined but non-genetic and is summarized in Table 1 [47,70,89].

Metreleptin provides a second, narrower example: replacement is effective in true leptin-deficiency or generalized lipodystrophy states but has limited rationale in common obesity, where circulating leptin is usually high and signaling is resistant. The clinical lesson is that a molecular diagnosis must identify a targetable signaling defect before a therapy is labeled precision pharmacotherapy [82].

## 14. Novel Mechanisms and Investigational Targets in Obesity Pharmacotherapy

The development of next-generation anti-obesity drugs is increasingly focused on enhancing pharmacodynamic precision, improving tolerability, and achieving sustained weight reduction through novel mechanisms (see also Section 11 and Table 3). These therapies employ diverse strategies that extend beyond traditional incretin pharmacology, including enzyme-targeted combinations such as EMP-16, a modified-release formulation pairing orlistat with acarbose. In the initial 26-week Phase II study, mean weight loss was 5.53–6.25% versus 0.83% with placebo; a later 6-month active-controlled study reported 7.73% with EMP-16 versus 5.78% with modified-release orlistat and 5.13% with conventional orlistat, with mainly mild, transient adverse events and no serious adverse events [48,137]. Mechanistically distinct agents such as LY3463251, a long-acting GDF15 analog and GFRAL/RET agonist, reduced food intake and appetite scores in humans but produced only modest body-weight reduction and dose-related nausea or emesis [50]. Peripherally restricted CB1R inverse agonists such as INV-202 achieved meaningful short-term weight loss in the Phase Ib study while avoiding the serious neuropsychiatric adverse effects, although this study could not exclude such risk [37]. In a 16-week Phase IIa trial, however, mild-to-moderate neuropsychiatric adverse events were more frequent with INV-202 (monlunabant) than with placebo and dose dependent [156]. Additional developmental targets include NK2R antagonists and oxytocin-pathway analogs [157,158,159]. Carbetocin showed nominal signals in CARE-PWS, but the confirmatory COMPASS PWS trial did not demonstrate efficacy versus placebo for hyperphagia [138,139]. Clinical translation likelihood (near term): dual GLP-1R/GCGR agonists such as survodutide and triple agonists such as retatrutide currently have the clearest pharmacodynamic-to-clinical continuity and late-phase momentum for obesity and metabolic liver disease [46,122,150].

## 15. Pharmacodynamic Correlates of Efficacy and Safety

Anti-obesity drug classes produce distinct metabolic and body-composition effects. GLP-1R, GIPR, and amylin-receptor agonism primarily reduces energy intake; GLP-1R and amylin-receptor activation also delay gastric emptying, although the dominant long-term weight effect is reduced intake rather than a consistently demonstrated rise in resting energy expenditure [11,34,35,112,133,134]. GCGR and MC4R engagement can increase substrate mobilization or energy expenditure, but the magnitude and tolerability depend on receptor balance and exposure [26,30,46,47,70,80,88,122,123,125,160,161]. Multi-receptor agonists produce large reductions in adiposity, but the preservation of lean mass is not established as a uniform class effect [76,80]. Body-composition data indicate that a substantial minority of the weight lost with high-efficacy incretin therapy is lean tissue, broadly comparable to the proportion seen with equivalent diet-induced weight loss; whether this translates into clinically meaningful loss of muscle function remains unresolved, and it is the explicit rationale for the body-composition-directed strategies discussed below [49,76,80]. By contrast, bimagrumab reduced fat mass by 20.5% while increasing lean mass by 3.6% at 48 weeks [49], whereas tirzepatide reduced fat mass by 33.9%, approximately three times the relative reduction in lean mass [76]; differences in population and weight-loss magnitude make this comparison descriptive only.

### 15.1. Efficacy Measured by Weight Loss Magnitude

The degree of weight loss achieved with anti-obesity medications is closely linked to mechanism, exposure, and tolerability [162]. Agents with predominantly peripheral mechanisms, such as orlistat, generally produce modest placebo-subtracted effects [59]. Centrally acting combinations and single-incretin agents, such as phentermine/topiramate and liraglutide, achieve intermediate mean reductions [64,68,69]. Semaglutide 2.4 mg produced a placebo-subtracted mean difference of 12.4 percentage points at 68 weeks in STEP 1, whereas tirzepatide produced placebo-subtracted differences of 11.9 to 17.8 percentage points at 72 weeks in SURMOUNT-1 [12,76]. SURMOUNT-5 provides the direct comparison and removes the need for cross-trial inference: tirzepatide produced −20.2% versus −13.7% with semaglutide 2.4 mg at 72 weeks, a randomized difference of 6.5 percentage points, substantially narrower than the 11.9–17.8 percentage-point spread suggested by placebo-anchored cross-trial comparison [119]. These comparisons are consistent with, but do not isolate, a multi-pathway contribution to efficacy, while cross-trial differences in population, baseline BMI, duration, titration, background lifestyle intervention, estimand, adherence, and exposure prevent simple receptor-based rankings.

### 15.2. Metabolic and Body Composition Benefits

Beyond weight reduction, anti-obesity medications can improve waist circumference and multiple cardiometabolic measures [6,45,59,64,76]. In a Phase II trial of patients with nonalcoholic steatohepatitis, semaglutide produced NASH resolution without worsening fibrosis in 59% of participants receiving the highest studied dose, compared with 17% receiving placebo; the trial did not establish that this effect was independent of weight loss [163]. Retatrutide combines GLP-1R, GIPR, and GCGR activity and has now been evaluated in Phase III. In TRIUMPH-1, mean weight reduction at week 80 was 19.0%, 25.9%, and 28.3% with 4, 9, and 12 mg respectively, compared with 2.2% with placebo, corresponding to a difference of approximately 26 percentage points at the highest dose [26,80,81]. These findings support substantial metabolic potential but do not by themselves establish superiority for liver or body-composition outcomes across drug classes.

Emerging therapies such as bimagrumab, an activin type II receptor (ActRII) inhibitor, represent a novel PD strategy to improve body composition by increasing lean mass while reducing fat mass. In a Phase II trial, bimagrumab led to a 6.5% reduction in body weight, a 20.5% decrease in fat mass, and a 3.6% increase in lean mass, highlighting its potential to preserve muscle during weight loss [49].

Improvements in cardiometabolic health accompany weight loss across several drug classes. GLP-1R agonists and tirzepatide improve glycemic measures, lipid profiles, and multiple cardiometabolic risk markers [45,75,76]. SGLT2 inhibitors provide cardiorenal and glycemic benefits in their approved populations, with modest secondary weight reduction [106,107]. Peripherally restricted CB1R inverse agonism has shown preliminary improvements in metabolic measures without serious neuropsychiatric events in a short Phase Ib study, but longer-term evidence is required [37,38]. These systemic effects are clinically important, yet they should be interpreted separately from direct receptor-level proof of a weight-loss mechanism. Cardiovascular outcome trials now allow this distinction to be tested directly. In SELECT, semaglutide 2.4 mg reduced major adverse cardiovascular events in patients with overweight or obesity and established cardiovascular disease but without diabetes (6.5% versus 8.0%; hazard ratio 0.80, 95% confidence interval 0.72–0.90), supporting a label extension for cardiovascular risk reduction [164]. In SURPASS-CVOT, tirzepatide was non-inferior but not superior to dulaglutide for major adverse cardiovascular events in type 2 diabetes with atherosclerotic disease (hazard ratio 0.92, 95% confidence interval 0.83–1.01), so cardiovascular benefit should not be presented as an established property of dual-receptor agonism [165]. Beyond cardiovascular endpoints, semaglutide achieved the resolution of steatohepatitis without worsening of fibrosis in the Phase III ESSENCE trial (62.9% versus 34.3% with placebo) [166], and tirzepatide has been approved for moderate-to-severe obstructive sleep apnoea in obesity evidence that incretin pharmacodynamics are now being tested against organ-specific rather than weight-only endpoints.

### 15.3. Dose–Response, Receptor Engagement, and Adaptation

Clinical efficacy reflects receptor pharmacology, pharmacokinetic exposure, and tissue engagement, but is ultimately bounded by the maximum exposure that can be sustainably tolerated: on-target gastrointestinal effects of GLP-1R agonism, and potentially heart-rate effects of GCGR agonism, cap dose escalation and thus achievable receptor activation (Section 16). Titration schedules for GLP-1R agonists and tirzepatide reflect exposure–response relationships constrained by pharmacodynamically mediated gastrointestinal tolerability. Gradual escalation permits more sustained receptor exposure while limiting nausea and vomiting [45,75,85]. Across approved agents, dose–response data show increasing weight loss with greater tolerated exposure (Table 1); however, approved maintenance doses reflect tolerability-limited titration rather than formally defined maximum tolerated doses, and exposure–response models are not available in comparable form across agents. Moreover, human receptor occupancy has not been measured in a standardized manner that permits direct ranking. Semaglutide and tirzepatide therefore should be described as providing prolonged exposure, through albumin-binding lipidation supporting once-weekly dosing, rather than as having proven higher GLP-1R occupancy than earlier analogs. Dose-related adverse effects follow predictable patterns: gastrointestinal symptoms increase with GLP-1R activation, whereas GCGR-containing agonists may also produce heart-rate signals [26,45,75,80,88].

STEP and SURMOUNT trials show that mean weight loss tends to approach a plateau during continued treatment, consistent with physiological compensation and the finite effect of a tolerated dose. This clinical pattern should not be interpreted as direct evidence of receptor desensitization. Partial weight regain after treatment withdrawal supports a chronic-treatment model of obesity pharmacotherapy [6,45,75].

Adaptive responses help explain the divergence between early and sustained effects of anti-obesity therapy. With GLP-1R agonists, attenuation of the gastric-emptying effect can occur during sustained exposure, whereas appetite and weight effects may persist; clinical plateauing is therefore not proof of receptor desensitization. Desensitization implies loss of drug effect at constant exposure, whereas compensation opposes a preserved effect; in SURMOUNT-4, tirzepatide withdrawal caused weight regain while continuation produced further loss, indicating persistent drug activity [75]. Weight-loss plateaus probably reflect a mechanisms supported by unequal evidence: reduced energy requirements, adaptive reductions in energy expenditure, and increased hunger are demonstrated in humans, although mainly after diet-induced rather than drug-induced weight loss [4]; tolerability limits on dose are evident in clinical trials [45,75]; and incomplete suppression of reward-driven eating remains inferred. Likewise, weight regain after treatment withdrawal supports defended-body-weight biology and the need for ongoing management, but it does not identify a single receptor-level mechanism [6,34,35,45,75].

A limitation of cross-agent pharmacodynamic comparison is that affinity, potency, and pathway data were generated in different receptor species, expression systems, cell backgrounds, assay formats, and exposure times. Table 2 therefore presents source-specific values and ranges rather than a direct potency ranking. Formal signaling-bias factors were reported only when matched pathway and reference-ligand data were available; otherwise, pathway-specific EC_50_/Emax, internalization, and tissue-distribution findings were used to avoid overinterpretation [52].

## 16. Pharmacodynamic Basis of Adverse Events

Adverse effects associated with AOMs often arise directly from their pharmacodynamic mechanisms, reflecting the physiological systems they target. For instance, GLP-1 receptor agonists such as liraglutide, semaglutide, and tirzepatide exert their anorectic effects by activating GLP-1 receptors in the hypothalamus and brainstem, particularly the area postrema, a chemoreceptor trigger zone involved in nausea and vomiting [12,76,85]. This central activation, combined with delayed gastric emptying, contributes to the high incidence of gastrointestinal side effects, including nausea, vomiting, and diarrhea. These effects are typically dose-dependent and may attenuate over time, but they remain a leading cause of treatment discontinuation [12,13,14].

Glucagon-receptor agonism in dual and triple agonists can enhance hepatic substrate mobilization and energy expenditure, but greater GCGR contribution may also produce heart-rate increases and other tolerability signals [26,46,80,88,161]. Effects on blood pressure are not uniform and should be reported from each clinical program rather than inferred from receptor identity alone. The contribution of GIPR agonism to gastrointestinal tolerability also remains uncertain; mechanistic hypotheses should not be presented as an established anti-nausea effect [34,35,54].

Other agents demonstrate adverse effects linked to off-target receptor activity. For example, setmelanotide, an MC4R agonist used in selected MC4R-pathway disorders including approved genetic indications and acquired hypothalamic obesity, has been associated with skin hyperpigmentation due to unintended activation of melanocortin-1 receptors, which regulate melanogenesis [47,70]. Similarly, earlier centrally acting CB1R antagonists such as rimonabant were withdrawn due to psychiatric side effects, including depression and suicidal ideation, attributed to CB1 blockade in the central nervous system [38]. In contrast, newer agents like INV-202 are designed to be peripherally restricted, intended to limit central CB1R engagement and thereby reducing neuropsychiatric risks [37].

These examples underscore the importance of receptor selectivity, tissue distribution, and signaling bias in shaping the safety profiles of AOMs. Strategies such as biased agonism, favoring beneficial intracellular pathways while minimizing adverse ones, and peripheral restriction are increasingly employed to enhance tolerability without compromising efficacy. Understanding these pharmacodynamic underpinnings is essential for anticipating adverse events, guiding dose titration, and informing the rational design of next-generation therapies. Tolerance/tachyphylaxis to monoaminergic agents (e.g., phentermine) has been documented clinically and is consistent with central catecholaminergic adaptation, reinforcing the preference for short-term use [55,56].

Beyond class-specific considerations, several pharmacodynamic principles underpin predictable safety profiles. GLP-1R activation produces dose-dependent gastrointestinal symptoms through vagal and brainstem pathways [34,35,45]. GCGR-containing agonists may increase heart rate, with the magnitude depending on receptor balance and exposure [26,46,80,88]. Setmelanotide can cause hyperpigmentation through melanocortin-receptor activity outside the intended MC4R pathway [47,70]. Amylin-receptor agonists and GDF15-GFRAL agonists can produce nausea or emesis through hindbrain satiation and aversive-response circuits [11,50,51,133]. Peripherally restricted CB1R inverse agonists are designed to limit the psychiatric toxicity associated with central CB1R blockade [37,38]. Post-marketing pharmacovigilance has since refined the safety framework for the incretin class in ways this section should reflect. Labeling for GLP-1 receptor agonists has been updated to address ileus and gastroparesis; delayed gastric emptying has prompted perioperative guidance on the risk of pulmonary aspiration during anesthesia; cholelithiasis and cholecystitis are recognized class effects attributable both to receptor-mediated effects on gallbladder motility and to rapid weight loss; regulatory reviews of suicidal ideation by the FDA and the European Medicines Agency did not establish a causal association; and an association with non-arteritic anterior ischaemic optic neuropathy has emerged as a signal under continuing evaluation [167]. Each of these fits the receptor-to-tissue framework used above, reflecting either on-target receptor engagement in a particular tissue or the physiological consequences of rapid fat loss.

## 17. Challenges in Anti-Obesity Pharmacotherapy

In addition to efficacy, tolerability and long-term safety remain critical challenges in obesity pharmacotherapy. Centrally acting agents such as phentermine/topiramate and naltrexone/bupropion, which modulate monoaminergic and GABA-mediated pathways, are associated with a range of neuropsychiatric and cognitive side effects. These include paresthesia, insomnia, anxiety, irritability, and memory impairment, particularly at higher doses or with prolonged use [64,67,97]. Such adverse effects can significantly impact adherence and are especially concerning in patients with underlying psychiatric comorbidities [97,98].

Moreover, highly specific pharmacodynamic targets can produce mechanism-linked side effects. Setmelanotide, an MC4R agonist approved for selected genetic MC4R-pathway disorders and acquired hypothalamic obesity, is associated with hyperpigmentation. This effect results from off-target activation of the melanocortin-1 receptor (MC1R), which regulates melanogenesis in the skin [47,70,71]. Monitoring is also required for depression/suicidality, sexual adverse reactions, and skin-pigmentation changes; acquired hypothalamic obesity additionally requires attention to adrenal function and sodium balance when clinically relevant [71].

To mitigate mechanism-linked adverse effects, newer agents are being designed with pathway preference or limited central exposure. INV-202 is a peripherally restricted CB1R inverse agonist intended to reduce brain exposure relative to earlier centrally acting CB1R antagonists such as rimonabant. In a 28-day Phase Ib trial, INV-202 produced approximately 3.3% mean weight loss versus a 0.5% gain with placebo and no serious adverse events; the study was too short and small to establish absence of neuropsychiatric risk [37]. Its reported clinical effects are consistent with peripheral CB1R modulation in adipose tissue, liver, and the gastrointestinal tract, but definitive receptor-occupancy and long-term safety data remain limited [37,38].

## 18. Conclusions and Future Perspectives

The pharmacotherapy of obesity has shifted from relatively nonspecific monoaminergic agents toward therapies that engage nutrient-responsive hormonal pathways. Semaglutide and tirzepatide exemplify high-efficacy appetite-centered therapy through prolonged GLP-1R or combined GIPR/GLP-1R activation [45,75,118]. Retatrutide remains investigational and adds GCGR activity, which may increase metabolic efficacy but also requires careful evaluation of heart-rate and tolerability effects [26,80]. These agents should not be grouped as if all enhance energy expenditure through glucagon signaling, because semaglutide and tirzepatide are not GCGR agonists.

This review has outlined receptor-level mechanisms of approved and investigational anti-obesity agents, emphasizing appetite regulation, glucose metabolism, energy balance, tissue access, and mechanism-linked adverse effects. Multi-receptor agonists can produce larger mean weight reductions than earlier single-target agents, but they do not eliminate physiological compensation, and their long-term comparative safety and durability remain incompletely defined. Clinical benefits include reductions in body weight, visceral adiposity, and several cardiometabolic risk markers.

Despite these achievements, several challenges remain. Obesity is a chronic, relapsing condition that requires long-term management. Sustaining efficacy over time, minimizing adverse effects, and improving adherence are critical goals. Long-acting formulations, the newly approved oral nonpeptide GLP-1RA orforglipron, and investigational pathway-preferential agonists such as ecnoglutide illustrate complementary strategies to improve convenience, tolerability, and long-term use [10,53,79].

Precision medicine remains a promising but incompletely validated frontier. Genomics, metabolomics, and phenotyping may eventually help identify subgroups that benefit from specific therapies, but most proposed biomarkers are not yet standards for common obesity. Setmelanotide has established pathway-defined indications in selected genetic MC4R-pathway disorders and acquired hypothalamic obesity, whereas metreleptin is used in true leptin-deficiency and generalized-lipodystrophy states [30,47,70,82,89]. Future studies should test biomarker-guided selection and dosing prospectively rather than assume clinical utility from exploratory signatures.

Expanding the pharmacodynamic arsenal beyond incretin pathways is also essential. Bimagrumab targets body composition and lean-mass preservation through ActRII blockade, whereas GDF15-GFRAL agonists are intended to provide an incretin-independent appetite-suppression pathway; for LY3463251, nausea and only modest human weight reduction remain important limitations [49,50]. Additionally, peripherally restricted agents like INV-202 are being designed to minimize central side effects while maintaining efficacy.

Finally, translating these clinical advances into public health impact will require addressing systemic barriers such as high drug costs, limited insurance coverage, and stigma surrounding obesity. Policy reforms and sustainable pricing models are needed to ensure equitable access to effective therapies.

In conclusion, the future of obesity pharmacotherapy lies in integrating pharmacodynamic innovation with long-term, individualized, and accessible care. The evidence behind these strategies differs. GLP-1R and GIPR/GLP-1R agonism are clinically validated by Phase III trials, as is MC4R agonism in pathway-defined indications. GCGR-containing and amylin-based combinations have Phase III efficacy data, but their long-term comparative safety and durability remain undefined. ActRII inhibition, GDF15 agonism, and peripheral CB1R inverse agonism remain early-stage, limited by small or short trials, modest weight loss, or neuropsychiatric signals. Next-generation therapy should build on validated mechanisms while testing the others prospectively.

## Data Availability

No new data were created or analyzed in this study. Data sharing is not applicable to this article.

## Supplementary Materials

The following supporting information can be downloaded at: https://www.mdpi.com/article/10.3390/pharmaceutics18101281/s1, Supplementary Material S1: Search strategy, inclusion criteria application, data-extraction fields, and evidence-tier definitions.

## Abbreviations

The following abbreviations are used in this manuscript:

AMPK	AMP-activated protein kinase
Aib	α-aminoisobutyric acid
ActRII	activin type II receptor
AMYR	amylin receptor
CB1R	cannabinoid type 1 receptor
cAMP	cyclic adenosine monophosphate
CTR	calcitonin receptor
EC_50_	half-maximal effective concentration
Fc	fragment crystallizable region (of an antibody)
GABA	gamma-aminobutyric acid
GPCR	G protein-coupled receptor
ERK	extracellular signal-regulated kinase
Gi/o	inhibitory G protein alpha subunits (Gi and Go)
Gs	stimulatory G protein alpha subunit
K_D (Kd)	equilibrium dissociation constant
Ki	inhibition constant
GLP-1R	Glucagon-like peptide-1 receptor
GIPR	Glucose-dependent insulinotropic polypeptide receptor
GCGR	Glucagon receptor
GDF15	Growth differentiation factor 15
IC_50_	half-maximal inhibitory concentration
LEPR	Leptin receptor
MC1R	melanocortin-1 receptor
MC4R	Melanocortin-4 receptor
MAOI	monoamine oxidase inhibitor
MEN 2	multiple endocrine neoplasia syndrome type 2
MTC	Medullary thyroid carcinoma
NFAT	nuclear factor of activated T cells
NE	Norepinephrine
NTS	nucleus tractus solitarius
nM	nanomolar
pM	picomolar
µM	micromolar
POMC	pro-opiomelanocortin
RAMP	Receptor activity-modifying protein
SGLT2	sodium–glucose cotransporter 2
GFRAL	GDNF family receptor alpha-like
RET	RET receptor tyrosine kinase
