# Supplementary material for: Progress in Anti-Obesity Drug Development: A Pharmacodynamics Perspective
Source: Pharmaceutics. 2026 Oct 9;18(10):1281. doi: 10.3390/pharmaceutics18101281 (PMC13708705; doi:10.3390/pharmaceutics18101281)
Supplement: Supplementary file 1 [file pharmaceutics-18-01281-s001.zip › pharmaceutics-4528525-supplementary.pdf]

Supplementary Material S1: Search Strategy, Inclusion Criteria Application, Data-Extraction Fields, and Evidence-Tier Definitions

1. **Search strategy**

   This is a narrative review. Sources were selected purposively against predefined criteria rather than by systematic screening. Therefore, no PRISMA flow diagram, record counts, or exclusion tallies were generated.

   - *Databases and period:* PubMed, Scopus, and Google Scholar, from inception to July 2026.
   - *Supplementary sources:* hand-searched reference lists, ClinicalTrials.gov, and regulatory resources (FDA labels, DailyMed, official announcements).
   - *General keywords:* "obesity pharmacotherapy," "GLP-1 agonists," "multi-receptor agonists," "pharmacodynamics."
   - *Targeted pharmacodynamic search:* each included agent or target name was combined with KD, Ki, EC50, IC50, Emax, receptor occupancy, cAMP, beta-arrestin, internalization, signaling bias, off-target, tissue distribution, or exposure-response. Primary receptor-pharmacology and human PK/PD studies were prioritized over narrative summaries.

2. **Inclusion criteria application**

   FDA-approved agents were included irrespective of the criteria. Emerging, investigational, repurposed, and regionally approved agents were included if they met at least one of the following:

   i. Approximately ≥5% weight loss in controlled human studies, the level regarded as clinically meaningful in obesity guidelines.
   ii. Mechanistic novelty (multi-agonism; amylin, ActRII, or GDF15 pathways), reflecting the pharmacodynamic focus.
   iii. Translational pharmacology data linking receptor engagement to clinical outcomes.

   Criteria were applied disjunctively across all drug classes. Agents meeting only (ii) or (iii) were retained despite weight loss below 5% (e.g., LY3463251, INV-202).
   Regulatory status as of July 2026 was classified as FDA-approved for obesity. (A), regionally approved for obesity (R), repurposed from another approved indication (Rp), or investigational (with highest phase).

3. **Data extraction field**

   For each included source, the following data were extracted: receptor species or subtype; expression system or tissue; assay type and readout; comparator; ligand; quantitative affinity or potency (KD, Ki, EC_{50}, IC_{50}); maximal response (Emax); trafficking or pathway data; tissue distribution; and documented secondary targets.

Values were not pooled across heterogeneous assays and were not imputed from a different ligand, assay, or receptor system. Ranges were used only for measurements that shared the same receptor species, expression system, and assay readout. Formal bias factors were reported only when matched pathways and a common reference agonist were available; otherwise, "pathway preference" is stated descriptively.

4. **Evidence-tier definitions**

Tiers describe the source and maturity of evidence. They are descriptive, not a formal GRADE rating, and follow the Oxford CEBM logic.

Tier 1: peer-reviewed confirmatory human data (Phase III RCTs, regulatory documents).

Tier 2: peer-reviewed exploratory human data (Phase II/Ib/IIa trials, substudies).

Tier 3: early-phase or mechanistic data (Phase I, receptor-level and animal studies).

Tier 4: non-peer-reviewed or secondary data (conference, company release, secondary review).
